# Health effects research and regulation of diesel exhaust: an historical overview focused on lung cancer risk

**DOI:** 10.3109/08958378.2012.691913

**Published:** 2012-06-04

**Authors:** Thomas W. Hesterberg, Christopher M. Long, William B. Bunn, Charles A. Lapin, Roger O. McClellan, Peter A. Valberg

**Affiliations:** 1 Navistar Inc., Chicago, Illinois, USA; 2 Gradient, Cambridge, Massachusetts, USA; 3 Lapin & Associates, Glendale, California, USA; 4 Toxicology and Human Health Risk Analysis, Albuquerque, New Mexico, USA

**Keywords:** Diesel exhaust, diesel emissions, lung cancer, new technology diesel exhaust (NTDE), epidemiology, mechanism, lung overload, elemental carbon, particulate matter, diesel particulate filter (DPF)

## Abstract

The mutagenicity of organic solvent extracts from diesel exhaust particulate (DEP), first noted more than 55 years ago, initiated an avalanche of diesel exhaust (DE) health effects research that now totals more than 6000 published studies. Despite an extensive body of results, scientific debate continues regarding the nature of the lung cancer risk posed by inhalation of occupational and environmental DE, with much of the debate focused on DEP. Decades of scientific scrutiny and increasingly stringent regulation have resulted in major advances in diesel engine technologies. The changed particulate matter (PM) emissions in “New Technology Diesel Exhaust (NTDE)” from today's modern low-emission, advanced-technology on-road heavy-duty diesel engines now resemble the PM emissions in contemporary gasoline engine exhaust (GEE) and compressed natural gas engine exhaust more than those in the “traditional diesel exhaust” (TDE) characteristic of older diesel engines. Even with the continued publication of epidemiologic analyses of TDE-exposed populations, this database remains characterized by findings of small increased lung cancer risks and inconsistent evidence of exposure-response trends, both within occupational cohorts and across occupational groups considered to have markedly different exposures (e.g. truckers versus railroad shopworkers versus underground miners). The recently published National Institute for Occupational Safety and Health (NIOSH)-National Cancer Institute (NCI) epidemiologic studies of miners provide some of the strongest findings to date regarding a DE-lung cancer association, but some inconsistent exposure-response findings and possible effects of bias and exposure misclassification raise questions regarding their interpretation. Laboratory animal studies are negative for lung tumors in all species, except for rats under lifetime TDE-exposure conditions with durations and concentrations that lead to'lung overload."The species specificity of the rat lung response to overload, and its occurrence with other particle types, is now well-understood. It is thus generally accepted that the rat bioassay for inhaled particles under conditions of lung overload is not predictive of human lung cancer hazard. Overall, despite an abundance of epidemiologic and experimental data, there remain questions as to whether TDE exposure causes increased lung cancers in humans. An abundance of emissions characterization data, as well as preliminary toxicological data, support NTDE as being toxicologically distinct from TDE. Currently, neither epidemiologic data nor animal bioassay data yet exist that directly bear on NTDE carcinogenic potential. A chronic bioassay of NTDE currently in progress will provide data on whether NTDE poses a carcinogenic hazard, but based on the significant reductions in PM mass emissions and the major changes in PM composition, it has been hypothesized that NTDE has a low carcinogenic potential. When the International Agency for Research on Cancer (IARC) reevaluates DE (along with GEE and nitroarenes) in June 2012, it will be the first authoritative body to assess DE carcinogenic health hazards since the emergence of NTDE and the accumulation of data differentiating NTDE from TDE.

## Introduction

Concern for the potential human carcinogenic hazard of exposure to internal-combustion engine exhaust, including diesel engine exhaust, arose more than a half century ago. These concerns prompted Kotin et al. (1954, 1955) to conduct mouse skin painting bioassays that yielded positive results for extracts of exhaust particulate matter from both gasoline and dieselfueledvehicles. Early epidemiological studies of railroad workers exposed to diesel exhaust (DE) yielded conflicting results (Hueper, 1956; Raffle, 1957; Kaplan, 1959). Since these earlier reports, more than 6000 papers have been published on a wide range of health endpoints investigated in the context of DE or DE constituent exposure (based on the toxicology subset of articles retrieved by searching on “diesel exhaust” in the US National Library of Medicine's PubMed online database). Mauderly (2001) has noted that the voluminous health effects literature on DE lags behind only that of cigarette smoke. Elevated exposures to airborne DE from diesel engines lacking modern aftertreatment systems have been linked with a variety of health concerns, including acute irritant effects (e.g. eye, throat, bronchial), respiratory symptoms (e.g. cough, phlegm, wheezing), immunologic effects (exacerbation of asthma and allergenic responses), lung inflammatory effects, cardiovascular health responses (e.g. thrombogenic and ischemic effects), and cancer (e.g. lung cancer).

The relationship between DE exposure and lung cancer risk has been a source of scrutiny by researchers and regulators over the last four decades, although there has been a shift in the last decade towards greater focus on non-cancer health endpoints such as cardiovascular and allergenic effects. Despite an extensive body of relevant studies that includes more than 50 epidemiologic analyses of occupationally exposed populations as well as a large number of chronic animal bioassays, scientific debate remains regarding the extent of the lung cancer risk posed by inhalation of occupational and environmental DE. Several published critical reviews and epidemiologic meta-analyses (e.g. HEI, 1995; Bhatia et al., 1998; Lipsett and Campleman, 1999; Lloyd and Cackette, 2001; Wichmann, 2007) have reached conclusions supportive of DE exposure increasing lung cancer risk, often citing epidemiologic studies showing a 20 to 50% increase in risk for workers exposed occupationally to DE relative to workers classified as unexposed. In addition, relying on historical DE studies (i.e. pre-2000 studies, predominantly of pre-1988 diesel engines), a number of cancer hazard assessments (e.g. NIOSH, 1988; IARC, 1989; IPCS, 1996; Cal EPA, 1998; NTP, 2000; US EPA, 2002) have concluded that elevated, long-duration exposures to DE, and specifically to diesel exhaust particulate (DEP), are likely linked with increased risk of lung cancer.

However, because of large uncertainties in exposure-response relationships observed in both human epidemiologic studies and laboratory animal studies, most authoritative bodies (e.g. IARC, 1989; IPCS, 1996; US EPA, 2002) have not made quantitative predictions of increased lung cancer risk as a function of DE exposure, either for workers or the general population. In addition, a number of other published assessments of the DE health effects evidence have concluded that neither the existing epidemiologic data nor the animal data are sufficient to reliably establish a causal link between DE exposure at either occupational or environmental levels and increased lung cancers (Stober and Abel, 1996; Muscat and Wynder, 1995; Cox, 1997; Morgan et al., 1997; Bunn et al., 2004; Hesterberg et al., 2005, 2006; Gamble, 2010; Gamble et al., 2012). As discussed later, these analyses have pointed to notable limitations in the existing health effects data, including the general absence of quantitative data on workers’ historical exposures to DE and the lack of human relevance of the species-specific lung overload mechanism underlying the tumorigenic effects observed in rats for protracted, elevated DE exposures.

The International Agency for Research on Cancer (IARC) will re-review DE in lune 2012 (along with GEE and some nitroarenes) and the US National Toxicology Program (NTP) recently nominated DEP for re-review in a future edition of the report on carcinogens. In light of these and other future DE evaluations, we offer perspective on the lengthy and voluminous record of research characterizing DE emissions, exposures, and potential health risks, focusing on the potential for DE to cause lung cancer. In contrast to GEE, which has been less studied from a health effects perspective (McDonald et al., 2007), there is a rich history of DE health effects research, encompassing a variety of approaches (e.g. *in vitro*, laboratory animal, humans exposed in chambers, and epidemiology of DE-exposed humans), engines, operating conditions, and health endpoints. Importantly, since the last major regulatory hazard assessment for DE conducted by the US Environmental Protection Agency (US EPA) in 2002, and especially since the 1988 IARC review, a number of additional studies have been published that bear on the relationship between DE exposure and lung cancer risk. Thus, the 2012 IARC assessment will be the first major carcinogenic hazard assessment to consider many of these new studies and data, with a possible NTP reevaluation of DEP to follow.

These hazard assessments will also be the first to address the emerging body of data related to what has been termed “new technology diesel exhaust” (NTDE) (Hesterberg et al., 2005). NTDE refers to the DE from current low-emission, advanced-technology diesel engines (both new and retrofitted) that incorporate multi-component emissions reduction systems (i.e. wall-flow diesel particulate filters (DPFs), diesel oxidation catalysts (DOCs), and ultra-low sulfur diesel (ULSD) fuel) designed to meet the US EPA 2007 particulate matter (PM) emissions standard of 0.01 g/bhp-hr. Similar to what has occurred over the past two decades, it is expected that diesel engine systems, fuels, and advanced-technology emission reduction system strategies will continue to evolve. As a result, other alternative advanced-technology diesel systems may be developed in the future that are capable of achieving a DE emissions profile for regulated and unregulated pollutants that is equivalent to NTDE.

As described in recent comprehensive reviews (Hesterberg et al., 2011; McClellan et al., 2012), there are now considerable emissions characterization data, as well as preliminary toxicological data, that show marked differences in emissions and toxicity between NTDE and “traditional diesel exhaust” (TDE) from pre-1988 diesel engines. NTDE is the product of paradigm-shifting technological innovation stimulated by the progressively more stringent DE emissions limits that have been implemented in the US and many other countries throughout the world, as well as the efforts of diesel engine manufacturers, emissions control technology companies (e.g. Corning, Johnson Matthey), government research laboratories, and academic researchers. In fact, among the major differences between DE at the time of the last IARC review in 1988 and today is that DE is now extensively regulated and major technological changes have occurred in diesel technology.

Our critical assessment is not intended to be another exhaustive review of DE emissions characterization data, exposure assessment studies, or health effects findings. A number of recent in-depth reviews on these topics are already available (Hesterberg et al., 2005, 2006, 2008, 2009, 2011; Burtscher, 2005; Maricq, 2007; Pronk et al., 2009; Mauderly and Garshick, 2009; Gamble, 2010). Instead, we aim to provide a roadmap of recent DE research and regulatory milestones of bearing to the DE-lung cancer question, directing scientists, regulators, and environmentalists to primary research articles as well as in-depth reviews. We provide a brief discussion and timeline of DE regulations in the United States because those regulations have had a major impact on reducing DE emissions and changing the composition of DE, and consequently, on any potential health risks. We document the extensive timeline of DE health effects research, focusing on more recent research milestones so as to critically examine the new pieces of scientific evidence that impact the assessment of the carcinogenic potential of TDE and NTDE.

## Background on DE regulatory history

Because diesel emission regulations have played a key role in stimulating technological innovation and ultimately to the emergence of NTDE (discussed in the next section), we begin with a brief regulatory overview. Table 1 focuses on US regulatory activities and provides a summary of key regulatory milestones, demonstrating how increasingly tighter emissions standards have culminated in today's stringent DE emissions limits. DE standards have also evolved in a similar fashion to stringent present-day emissions limits in other countries worldwide, with many countries adopting European Union (EU) diesel standards (more information on international diesel emissions standards can be found at: http://www.dieselnet.com/standards/). EU nations are currently phasing in Euro VI heavy-duty diesel engine (HDDE) requirements (e.g. for steady-state test procedures, 0.01 g/kWh for PM and 0.4g/kWh for NO_x_; UNECE, 2012) that were approved in December 2008 by the European Parliament and are approximately equivalent to the US EPA 2010 on-road HDDE emissions limits.

**Table 1:** Key regulatory actions affecting diesel engine exhaust in the United States

Year	Event
1968	First “smoke standard” promulgated in the US for onroad HDDE (33 FR 8304, June 4, 1968)
1970	Clean Air Act of 1970 provide US EPA with authority to issue National Ambient Air Quality Standards (NAAQS), as well as provisions for regulating diesel engines and fuels. (42 U.S.C. §7401 et seq. (1970))
1971	Issuance of initial NAAQS for criteria air pollutants (PM, CO, NO_x_, SO_2_, HC, and O_3_) (36 FR 8186, April 30, 1971)
1974	US EPA implementation of first US CO standard and a combined HC and NO_x_ standard for onroad HDDE
1977	US EPA issues precautionary notice of the mutagenicity of organic solvent assays of diesel exhaust particles in bacterial assays (November 4, 1977)
1979	US EPA, along with the US Department of Energy and the Department of Transportation, request that the National Research Council conduct an evaluation of the potential health impacts associated with prospective widespread use of diesel-powered light-duty vehicles in the United States
	US EPA implementation of new HC standard for on-road HDDE (while retaining the combined HC+NO_x_ standard)
1982	US EPA introduction of first on-road diesel engine PM emissions standard (light-duty diesel cars and trucks, but not HDDE) (45 FR 14496, March 5, 1980)
1985	US EPA implementation of new NO_x_ standard (10.7 g/bhp-hr) for on-road HDDE, and elimination of combined HC+NO_x_ standard. (50 FR 10606, March 15, 1985)
1987	US EPA reduces PM standards to 0.2 g/mile and 0.26 g/mile for light-duty diesel cars and trucks, respectively (47 FR 54250, December 1, 1982)
	US EPA replaces TSP-based PM NAAQS with PM_10_ standards (52 FR 24634, July 1, 1987)
1988	US EPA introduction of first PM standard for on-road HDDE (0.6 g/bhp-hr)
1990	State of California, under the Safe Drinking Water and Toxic Enforcement Act of 1986 (Proposition 65) identifies diesel exhaust as a chemical “known to the State to cause cancer”
	US EPA implements reduced on-road HDDE NO_x_ standard of 6.0 g/bhp-hr
1991	US EPA implements reduced PM standard of 0.25 g/bhp-hr for HDDE in trucks and urban buses
	US EPA implements reduced on-road HDDE NO_x_ standard of 5.0 g/bhp-hr
1993	US EPA implements reduced PM standard of 0.1 g/bhp-hr for HDDE in urban buses
	US EPA regulations for sulfur (500 ppm limit) and aromatic hydrocarbons (no more than 35% by weight) in highway diesel fuel go into effect
1994	US EPA implements reduced PM standards of 0.1 g/bhp-hr and 0.07 g/bhp-hr for on-road HDDE in trucks and urban buses, respectively
	US EPA establishes first emissions standards (Tier 1 emissions standards for CO, HC, PM, NO_x_, and smoke emissions) for non-road diesel engines at or above 37 kW (59 FR 48472, September 21, 1994)
	US EPA Tier 1 standards for light-duty vehicles go into effect, with a phase-in implementation schedule of 1994–1997
1996	US EPA implements reduced PM standard of 0.05 g/bhp-hr for on-road HDDE in urban buses
1997	US EPA finalizes rulemaking establishing new emission standards for model year 2004 and later truck and bus HDDE, targeting
	NO_x_ and non-methane hydrocarbons (NMHC) using two alternative standards (either a combined NO_x_+NMHC limit of 2.4 g/bhp-hr, or a NO_x_ limit of 2.5 g/bhp-hr and a NMHC limit of 0.5 g/bhp-hr) (62 FR 54694, October 21, 1997)
	US EPA issues first fine particulate matter (PM_2.5_) NAAQS (62 FR 38652, July 18, 1997)
1998	US EPA implements reduced NO_x_ standard of 4.0 g/bhp-hr for all on-road HDDE
	US EPA finalizes first emission standards for locomotives and puts in place a three-tiered system for regulating engines manufactured between 1973 to 2001, 2002 to 2004, and post-2005 beginning in 2000 (63 FR 18978, April 16, 1998)
	US EPA finalizes more stringent emission standards (Tiers 2 and 3) for NO_x_, HC, and PM from new non-road diesel engines, including the first set of standards for non-road diesel engines below 37 kW. (63 FR 56967, October 23, 1998)
1999	US EPA issues first emissions standards for commercial marine diesel engines at or above 37 kW, establishing Tier 1 (voluntary NO_x_ approach) and Tier 2 (for combined HC + NO_x_, PM, and CO) emission standards for new Category 1 and 2 marine diesel engines smaller than 30 liters per cylinder. (64 FR 73300, December 29, 1999)
2000	US EPA promulgates the first emission standards for marine diesel engines to take effect between 2004 and 2007 (Proposed Rule – 65 FR 76797 – December 7, 2000)
	US EPA lists diesel exhaust as a “mobile source air toxic”
2001	US EPA finalizes the “2007 Heavy-Duty Highway Rule,” establishing updated emission standards for 2004 and later heavy-duty highway engines and vehicles and highway diesel fuel sulfur control requirements (ultra-low sulfur diesel fuel with sulfur levels at or below 15 ppm) (66 FR 5002, January 18, 2001)
	MSHA publishes final rule establishing DPM concentration limits (interim concentration of 400 μg of total carbon per m^3^ to go into effect in July 2002, and a final concentration limit of 160 μg of total carbon per m^3^ to go into effect in January 2006) for under-ground metal and non-metal miners (66 FR 5706, January 19, 2001)
2002	US EPA finalizes first emissions standards (for combined HC + NO_x_, PM, and CO) for recreational marine diesel engines over 37 kW (67 FR 68242, November 8, 2002)
2003	US EPA issues final rule establishing near-term, Tier 1 emission standards for NO_x_ for new (2004 and later) commercial marine diesel engines (Categories 1, 2, and 3) that will be installed on vessels flagged or registered in the United States (68 FR 9746, February 28, 2003)
2004	US EPA adopts Clean Air Nonroad Diesel Final Rule, putting in place a comprehensive program to reduce NO_x_ and PM emissions by more than 90 percent from non-road diesel engines that includes Tier 4 emissions standards and the first regulations to reduce the allowable sulfur content (by more than 99 percent) in diesel fuels used in non-road diesel engines, locomotives, and marine vessels (68 FR 38958 – June 29, 2004)
	1997 NO_x_/NMHC HDDE emissions standards go into effect (62 FR 54694, October 21, 1997)
	US EPA Tier 2 standards for light-duty vehicles go into effect, tightening the previous Tier 1 emissions limits and establishing consistent emission standards regardless of vehicle weight and fuel type, with a phase-in implementation schedule of 2004–2009 (see 1998)
2005	MSHA issues final rule with revisions to its DPM concentration limits for underground metal and non-metal miners, replacing the interim DPM concentration limit with a permissible exposure limit (PEL) of 308 μg/m^3^ measured as elemental carbon (70 FR 32868, June 6, 2005)
2006	Effective year of US EPA's 2001 standard for highway ultra-low sulfur (15 ppm) diesel fuel (ULSD) (66 FR 5002, January 18, 2001)
	MSHA publishes a final rule phasing in the DPM final concentration limit of 160 (Total Carbon) μg/m^3^ over a two-year period based on feasibility, with a final compliance date of May 20, 2008 (71 FR 28924, May 18, 2006)
	US EPA reduces the 24-h PM_2.5_ NAAQS from 65 μg/m^3^ to 35 μg/m^3^ (71 FR 61144, October 17, 2006)
2007	US EPA 2001 PM emissions standard for new heavy-duty engines of 0.01 g/bhp-hr goes into effect; beginning of phase-in of updated standards for NO_x_ and NMHC of 0.20 g/bhp-hr and 0.14g/bhp-hr (see 2001)
	Non-road diesel engines, including locomotives and smaller marine engines, now required to use low sulfur (500 ppm) diesel fuel (see 2004)
2008	US EPA finalizes more stringent emissions standards for locomotives and marine diesel engines, including Tier 3 and Tier 4 standards intended to reduce PM and NO_x_ emissions by 80–90% and the first national emission standards for existing marine diesel engines (73 FR 25098, May 6, 2008)
2010	US EPA 2001 updated NO_x_ and NMHC emissions standards to be in full effect (see 2001)
	US EPA finalizes rule adding two new tiers of Category 3 (C3) marine diesel engine emission standards (Tier 2 and Tier 3 standards for NO_x_, HC, and CO) and revising its standards for marine diesel fuels produced and distributed in the United States (75 FR 22896, April 30, 2010)
	Effective year for requirement that non-road diesel engines use ultra-low sulfur (15 ppm) diesel fuel (see 2004)
2012	Effective year for requirement that locomotives and smaller marine engines use ultra-low sulfur (15 ppm) diesel fuel (see 2004)

Notes: For those regulatory activities where specific regulatory citations could not be identified, US EPA (1997, 2002) are the information sources.

**Figure 1:**
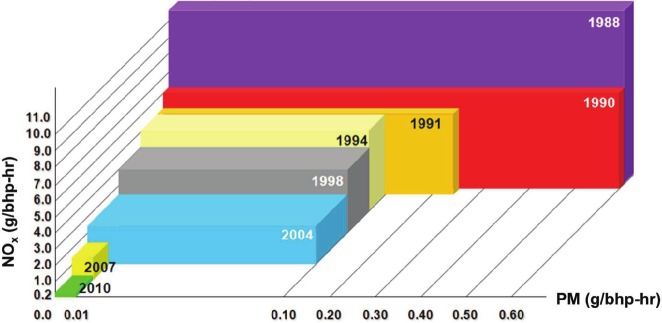
Evolution of US heavy-duty diesel engine on-road emissions standards, expressed as grams PM or NO_x_ emitted per brake-horsepower-hour (g/bhp-hr). Note that in 2004 two alternative standards were implemented: either a combined NO_x_+NMHC limit of 2.4 g/bhp-hr, or a NO_x_ limit of 2.5 g/bhp-hr and a NMHC limit of 0.5 g/bhp-hr. See Table 1 for additional details and citations for the emissions standards. (See colour version of this figure online atwww.informahealthcare.com/iht)

As shown in Table 1, US EPA exercised the authority given to it in the Clean Air Act of 1970 (42 U.S.C. §7401 et seq. (1970)) when it implemented the first emissions standards for carbon monoxide (CO), nitrogen oxides (NO_x_), and hydrocarbons (HC) in HDDE emissions in 1974 (US EPA, 2002). However, the PM emissions from diesel engines were largely unregulated in the US until early reports (NY Times, 1977; Huisingh et al., 1978) of the mutagenicity of organic solvent extracts of DEP set in motion a standard-setting process in the 1980s that ultimately resulted in the current stringent PM standard of 0.01 g/bhp-hr for on-road HDDEs. As shown in Figure 1, PM and NO_x_ emissions for on-road HDDEs have been reduced by approximately 98% since 1988 (for PM, from 0.60 to 0.01 g/bhp-hr; for NO_x_, from 10.7 to 0.2 g/bhp-hr; see Table 1 for references to the emissions standards). Although the first emissions standards for non-road diesel engines were not established by US EPA until 1994, progressively more stringent standards have also been implemented in the US in recent years for non-road engines, as well as for locomotives and marine diesel engines (US EPA, 2002). Parallel to the efforts to tighten emission standards for regulated pollutants, US EPA has also mandated fuel requirements that have greatly reduced the sulfur content of diesel fuels for on-road and off-road vehicles (Table 1). Although US EPA requirements stipulated that ULSD be the dominant diesel fuel produced in the US after June 2006, it was not until December 2010 when nationwide retail outlets outside of California (note that California had an earlier deadline of September 2006) no longer had the option of selling either low sulfur diesel or ULSD and could only sell ULSD (http://www.clean-diesel.org/).

In addition to diesel emissions standards and fuel requirements, US EPA (and other international agencies) have also implemented increasingly stringent air quality standards for PM that have implications for diesel emissions. As shown in Table 1, the PM National Ambient Air Quality Standards (NAAQS) have evolved to address progressively smaller size fractions - from the original total suspended particulate (TSP) indicator in 1971, to a PM_10_ indicator focused on particles less than 10 micrometers in diameter in 1987, to the current PM_25_ indicator focused on particles less than 2.5 urn in diameter in 1997 (US EPA, 2009). Given that DEP from TDE consists primarily of PM_2__5_, this has had the effect of focusing additional regulatory scrutiny on diesel engine emissions.

In addition to US EPA, the US Mine Safety and Health Administration (MSHA) has also adopted more stringent standards to control diesel emissions in underground mines (Table 1). As part of its 2006 final rule addressing diesel particulate matter (DPM) exposures of underground metal and non-metal miners (71 FR 28924), MSHA adopted a phased schedule for meeting the current permissible exposure limit (PEL) of 160 (total carbon) u.g/m^3^ by May 2008.

## Development of diesel engine technology and changes in diesel exhaust emissions

Invented by Rudolf Diesel in the 1890s, diesel engines are a specialized type of internal-combustion engine. Diesel engines use high pressure, rather than an electrical spark, to ignite hydrocarbon fuel vapors. Similar to other hydrocarbon combustion processes, the main combustion products in diesel engine exhaust are carbon dioxide (CO_2_) and water (H_2_O). However, DE also contains a highly-complex mixture of hundreds of chemicals, which are found in low concentrations in both particulate and gaseous form. As discussed below, a wealth of DE emissions characterization data are now available to support major differences in emissions levels and the composition of TDE from older, traditional diesel engines with NTDE from new and retrofitted engines utilizing multi-component emissions reduction systems (i.e. wall-flow DPFs, DOCs, and ULSD fuel) (Hesterberg et al., 2011; McClellan et al., 2012). Note that these new technologies were mandated after 2006 for new on-road HDDEs by the tightened PM emissions standard of 0.01 g/bhp-hr in US EPA's 2007 Heavy-Duty Highway Rule (66 FR 5002), and consequently, we often refer to the NTDE from post-2006 on-road HDDEs.

### Traditional diesel exhaust (TDE) composition

TDE is well-known to consist of three basic components, namely, (1) respirable-size aggregates of elemental carbon (EC) particles, with (2) coatings of organic matter and sulfates, accompanied by (3) a mixture of gas and vapor phases that include mainly nitrogen gas (N_2_), oxygen gas (O_2_), H_2_O, CO_2_, CO, NO_x_, sulfur dioxide (SO_2_) and other sulfur compounds, and low-molecular-weight HC (Hesterberg et al., 2005; US EPA, 2002). It contains a number of other compounds the US EPA has characterized as hazardous air pollutants (HAPs), including formaldehyde, acetaldehyde, acrolein, benzene, 1,3-butadiene, and polycyclic aromatic hydrocarbons (PAHs) (US EPA, 1995, 2002). The various DE constituents are known to vary in composition and concentration depending on engine type, fuel type, and operating conditions; detailed breakdowns of DE composition and emissions factors can be found in Schuetzle (1983), Johnson (1988), IARC (1989), US EPA (2002), McDonald et al. (2004a), Hsu and Mullen (2007), and Hesterberg et al. (2008). In addition, diesel emissions have been constantly evolving over time, due to the progressively more stringent regulations, continuous improvements to the internal design of the diesel engine, and the commercialization of aftertreat-ment technologies. In other words, improvements in diesel engine technologies and adoption of aftertreatment technologies contributed to DE emissions reductions prior to the more widespread adoption of the combination of new technologies (wall-flow DPFs, DOCs, and ULSD fuel) among post-2006 on-road HDDEs and retrofitted HDDEs that define NTDE.

DEP has been the primary focus of DE-related health concerns (see reviews by Maricq, 2007; Burtscher, 2005), and considerable effort has been directed to understanding the properties of DEP, also sometimes referred to as DPM. Even when the substantial mass of CO_2_ and water vapor in DE is disregarded, DEP generally contributes less than 1% of the total mass of diesel-fuel combustion products, including for older diesel engines operated using high-sulfur diesel fuel (Mauderly and Garshick, 2009). DEP can, however, be a significant contributor to ambient PM levels; for example, source apportionment data indicate that diesel combustion sources can contribute on the order of 10% of urban fine PM levels in some US cities (Díaz-Robles et al., 2008; Martello et al., 2008; Sarnat et al., 2008). DEP from traditional (pre-1988) diesel engines is dominated by submicron particles that consist of EC cores and adsorbed organic compounds, along with small amounts of sulfate, nitrate, metals, and other trace elements (US EPA, 2002). DEP-adsorbed organics have been shown to include chemical mutagens such as PAHs, nitro-PAHs, and oxidized PAH derivatives, although as discussed later, studies have demonstrated that these organic DEP constituents are only poorly bioavailable in aqueous-based lung fluids.

### The emergence of new technology diesel exhaust (NTDE)

**Figure 2:**
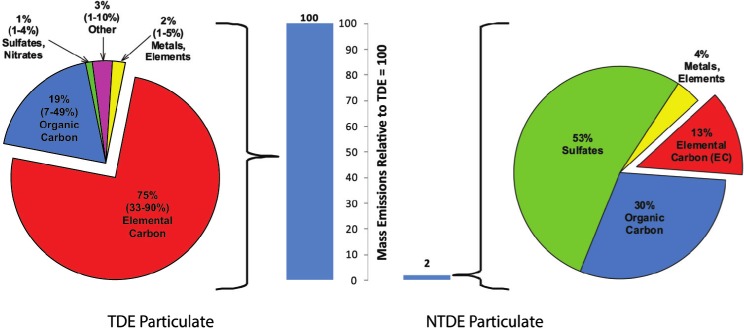
Chemical compositions of PM in NTDE (data from Khalek et al., 2011; based on averaged data for four 2007-model-year heavy-duty diesel engines, including three equipped with a diesel oxidation catalyst (DOC) and a catalyzed diesel particulate filter (c-DPF), and engine equipped with an exhaust diesel fuel burner and c-DPF) versus TDE (data from US EPA, 2002; for 1990s-era diesel engine technology) from heavy-duty diesel engines. PM mass emissions bars for NTDE and TDE derived from data compiled in Hesterberg et al. (2008) for diesel school buses with and without catalyzed DPFs (used in conjunction with ULSD), respectively. Note that there can be variability in PM emissions for diesel engine technologies considered to emit NTDE and TDE, such that data from other studies may differ from those in the figure. In general, as illustrated in these comparisons, not only is less PM emitted in NTDE on a per mile basis, but the emitted PM differs in composition from the PM emitted in TDE. (See colour version of this figure online atwww.informahealthcare.com/iht)

Stimulated by the progressively more stringent DE emissions limits over the last two decades, major advances in diesel engine technology have resulted in substantial reductions in DEP mass emissions and significant changes in DEP composition, as well as reduced emissions of gaseous constituents. Figure 2 illustrates the major differences in DEP mass emissions and composition between TDE and NTDE, recognizing that emissions from specific engines/technologies can vary depending on a number of factors including engine specifications, fuel, operating cycle, sampling techniques, etc. As noted earlier, NTDE refers to the exhaust from modern new and retrofitted advanced diesel engines that incorporate multi-component aftertreatment systems, including wall-flow DPFs, DOCs, and ULSD fuel, designed to meet the tightened US EPA PM emission standard for 2007 on-road HDDEs. Although the DPF is widely recognized as the centerpiece of modern aftertreatment systems needed to meet today's stringent PM emissions limits (Maricq, 2007), the transition to ULSD was also a key event in the emergence of NTDE since ULSD is essential to the proper functioning of DPFs. The end-product of US EPA diesel fuel regulations, as well as technological innovation in refinery processes, ULSD at 0.0015% or less sulfur is indeed radically different from diesel fuel in the 1980s when typical sulfur contents were in the range of 0.23 to 0.28% (US EPA, 2002). The transition to ULSD has been linked with noteworthy air quality improvements; for example, some studies have reported significant reductions in particle number concentrations in heavily-trafficked urban areas coinciding with the introduction of ULSD (Jones et al., 2012; Wahlin et al., 2001).

As illustrated by Figure 2, consistent PM mass reductions of >90% have been observed for NTDE from retrofitted and post-2006 on-road HDDE engines, compared to DE from post-1990 and post-2000 engines, let alone TDE from pre-1988 engines (Khalek et al., 2011; Herner et al., 2009; Biswas et al., 2009a). In addition, modern aftertreatment devices such as DOCs and DPFs have altered DEP composition, with Khalek et al. (2011) reporting characterization data from the Advanced Collaborative Emissions Study (ACES; discussed in detail later) showing the mass composition of DEP in NTDE to be dominated by sulfates (53%) and organic carbon (OC; 30%), rather than the EC typical of TDE (13% for NTDE versus 33 to 90% in TDE, depending on operating conditions). Khalek et al. (2011), as well as other studies (e.g. Biswas et al., 2009a; Liu et al., 2008, 2010; Thalagavara et al., 2005; Tang et al., 2007), demonstrate that the EC particles characteristic of TDE are largely eliminated from NTDE. The shift from a dominant insoluble EC fraction to a composition with major soluble sulfate and OC fractions has important toxicological implications, because as discussed later, it is the insoluble EC fraction of DEP that has been linked with tumor formation in rats *via* a lung overload mechanism.

**Figure 3:**
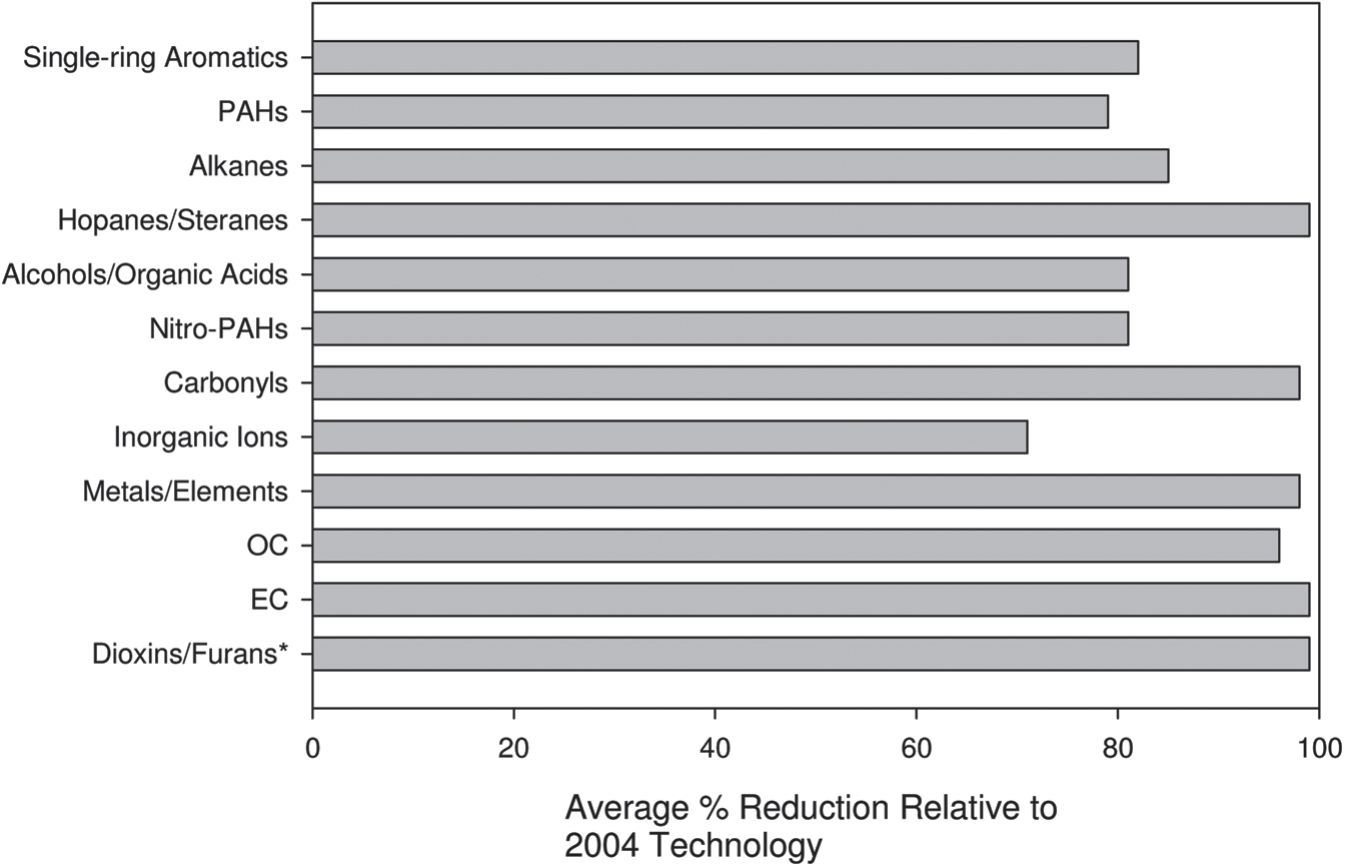
Average % reductions for DEP chemical classes relative to 2004 diesel technology engines for ACES testing of four post-2006 technology diesel engines (data from Khalek et al., 2011). ACES testing for 12 repeats of 16-h transient cycle developed at West Virginia University that covers a complete engine operation with active regeneration events. *Reductions in dioxins/furans are for comparison with 1998 technology engines.

As recently reviewed in Hesterberg et al. (2011) and McClellan et al. (2012), the changed chemical and physical properties of NTDE from retrofitted and post-2006 on-road HDDE engines (in contrast to TDE from pre-1988 engines, as well as transitional DE from post-1990 and post-2000 engines) are now well-documented in a series of recent DE characterization studies (see in particular Biswas et al., 2008, 2009a, 2009b; Herner et al., 2009, 2011; Hesterberg et al., 2008; Hu et al., 2009; Khalek et al., 2011; Laroo et al., 2011; Liu et al., 2008, 2010, 2011; Maricq, 2007; Pakbin et al., 2009; Ullman et al., 2003). As illustrated in Figure 3, these studies demonstrate major emissions reductions across a variety of DE chemical classes in NTDE, including PAHs, nitro-PAHs, carbonyls, metals, dioxins/furans, and both EC and OC (e.g. 71-99%, just between 2004 and 2007). Of particular relevance to the potential carcinogenicity of DEP in NTDE, recent studies have reported >99% removal efficiencies for a number of PAH and nitro-PAH compounds in NTDE compared to 1990s/2000s technology engines (Khalek et al., 2011; Pakbin et al., 2009).

Studies have demonstrated significant reductions in not only DEP species but also gaseous DE species. For example, relying upon emissions data from 25 studies of transit buses, school buses, refuse trucks, and passenger cars, Hesterberg et al. (2008) documented substantial reductions in the levels of carbon monoxide, total HC, non-methane hydrocarbons (NMHC), formaldehyde, benzene, acetaldehyde, and PAHs in NTDE. Ullman et al. (2003) reported that 21 of the 41 “toxic air contaminants” (TACs) listed by the California Air Resources Board (CARB) as being present in TDE could not be detected in exhaust from an advanced technology diesel engine equipped with a catalyzed particulate filter. In contrast to CO, various HC, and aldehydes, there is evidence that gaseous NO_x_ species are not dramatically reduced in NTDE from new or retrofitted on-road HDDEs meeting the 2007 US EPA PM emissions standard. For example, although Khalek et al. (2011) reported that NO_x_ emissions for the four ACES 2007-model-year HDDEs were on average 9% lower than the 2007 US EPA NO_x_ standard, NO_2_ emissions were on average 1.3 and 2.3 times higher than those from 1998 and 2004 technology engines. Herner et al. (2009) further demonstrated the small effect of DPFs on total NO_x_ emissions. Beginning with the 2010 model year, all new on-road HDDEs are required to have NO_x_ exhaust control technology - e.g. selective catalytic reduction-urea (SCR-urea) systems and/or advanced exhaust gas recirculation (EGR) - that will reduce NO_x_ emissions down to the stringent standard of 0.2 g/bhp-hr.

**Figure 4:**
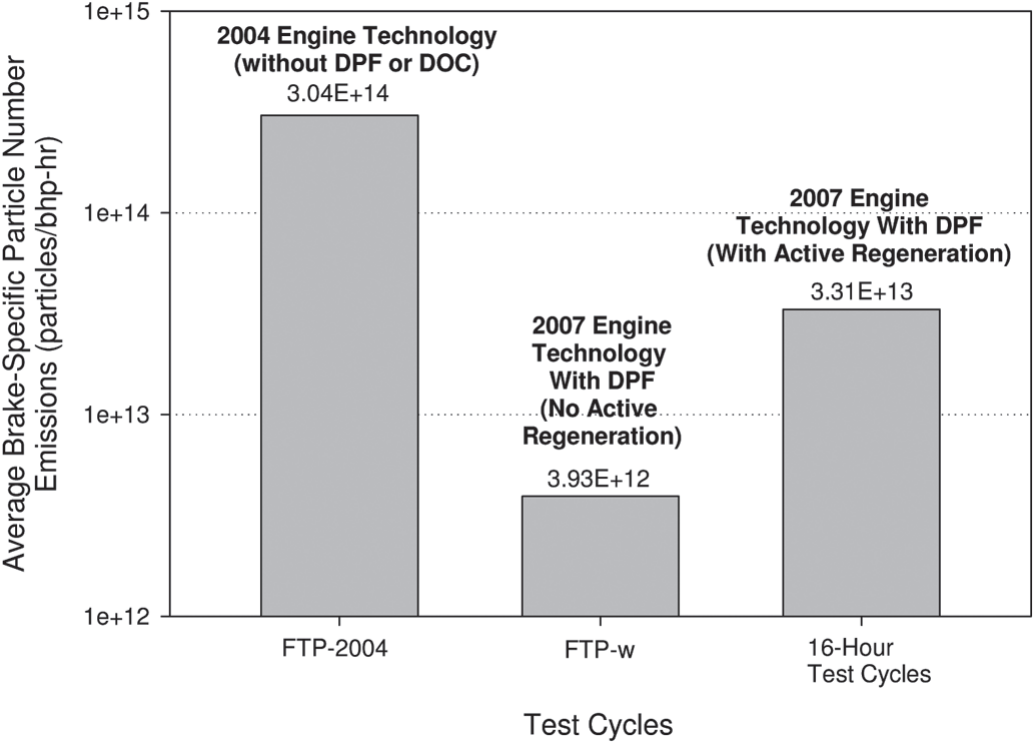
Average particle number emissions (note the logarithmic scale) for 2007 ACES engines (with and without c-DPF regeneration) versus a 2004 technology engine. As discussed in Khalek et al. (2011), data for the 2007 ACES engines were based on 12 repeats of the 20-min federal test procedure transient cycle (FTP) or 12 repeats of the 16-h cycle, each for all four ACES engines and for sampling from an unoccupied animal exposure chamber set up on a constant volume sampler (CVS). Data for the 2004 technology engine were based on six repeats of the FTP transient cycle from a full flow CVS. All data are reported on a brake-specific emissions basis, which is defined by Khalek et al. (2011) as the total emissions during a test interval over the work expressed in brake horsepower-hour.

As major reductions in DEP mass emissions were achieved with advanced diesel engine technologies and aftertreatment devices, it was hypothesized in the 1990s that the large reductions in “condensation surfaces” may promote particle nucleation and result in significant increases in diesel nanoparticle emissions (Bagley et al., 1996; Kittelson, 1998). Diesel nanoparticles (also commonly referred to as ultrafine particles, and generally defined as particles with diameters of 100 nm and smaller) have been the subject of many recent DE characterization studies, and we now have a better understanding of diesel nanoparticle emissions in TDE and NTDE (as reviewed in Hesterberg etal., 2011; McClellan et al., 2012; Maricq, 2007; Burtscher, 2005; Kittelson, 1998). In particular, as reflected in Figure 4 for the ACES testing, there is good quantitative evidence from a number of recent studies of the effectiveness of catalyzed DPFs (c-DPFs) for removal of DEP nanoparticle emissions (Khalek et al., 2011; Biswas et al., 2008; Herner et al., 2011; Kittelson et al., 2006; Holmén and Ayala, 2002; Holmén and Qu, 2004; Nylund et al., 2004; Liu et al., 2003; Ayala & Herner, 2005; Bosteels et al., 2006; Frank et al., 2007).

There is also evidence from some studies that certain aftertreatment configurations, in particular those containing catalyzed surfaces (e.g. c-DPFs, DOCs, SCR-urea systems), and operating conditions may promote formation of nucleation-mode particles in NTDE (Biswas et al., 2008; Herner et al., 2011; Kittelson et al., 2006; Vaaraslahti et al., 2004; Swanson et al., 2009). Study findings suggest that the formation potential of nucleation-mode particles in NTDE is dependent on a number of factors, including aftertreatment specifications (e.g. catalytic loading, sulfur exposure history), operating conditions (driving cycle, and more specifically, exhaust temperature and load), and fuel and engine oil sulfur content (Herner et al., 2011). Importantly, data are emerging that show large differences in the composition of DEP nanoparticles in NTDE versus TDE, shifting from a HC-rich composition for nanoparticles in TDE to a sulfate-rich composition for nanoparticles in NTDE (Maricq, 2007; Biswas et al., 2009a; Herner et al., 2011; Grose et al., 2006; Kittelson et al., 2006; Burtscher, 2005; Tobias et al., 2001). Although a possible role of DEP nanoparticles in DE carcinogenic potential has not been directly investigated, it has been speculated that the sulfate-rich composition of NTDE nanoparticles will lead to reduced toxicity due to the low intrinsic toxicity of highly-soluble sulfate particles (Herner et al., 2011; Hesterberg et al., 2011; Grose etal., 2006).

### Concluding remarks on changes in DE emissions

In conclusion, there is now a sizeable body of data showing that NTDE is strikingly different in chemical and physical properties from DE emitted by pre-1988 (i.e. pre-regulation of DEP emissions) diesel engines, as well as post-1990 and post-2000 engines lacking modern aftertreatment components (Hesterberg et al., 2011; McClellan et al., 2012). Despite the surge in emissions characterization data for NTDE, there remain some data gaps and uncertainties, in particular involving nanopar-ticle emissions. For example, there is growing evidence demonstrating the effectiveness of DPFs in removing diesel nanoparticles (e.g. Khalek et al., 2011; Biswas et al., 2008; Herner et al., 2011; Kittelson et al., 2006; Holmén and Ayala, 2002; Holmén and Qu, 2004; Nylund et al., 2004; Liu et al., 2003; Ayala & Herner 2005; Bosteels et al., 2006; Frank et al., 2007), but additional study is needed to characterize the range of conditions that may promote formation of nucleation-mode particles in NTDE and the health-effect implications of DEP nanoparticle emissions in NTDE. In these studies, it will be important to account for potential nanoparticle artifacts arising from unrealistic experimental conditions, such as from dilution rates, dilution ratios, temperatures, residence times, and relative humidities (Hesterberg et al., 2011).

## Progress in DE exposure assessment

Parallel to the recent advances in the characterization of DE emissions, recent studies have also attempted to improve our understanding of DE exposures in both occupational and environmental settings. As discussed in prior reviews (Schauer, 2003; US EPA, 2002), DE exposure assessment has proven to be a challenging exercise, given the lack of indicator chemicals unique to the complex DE mixture versus other combustion sources. Studies have relied upon a variety of different surrogates for DE and DEP exposure concentrations, including respirable PM, EC, OC, total carbon (TC), andNO_2_. Since the 1990s when it was identified as a more specific and sensitive surrogate of DE, EC has gained increasing use as a preferred surrogate measure of DEP exposure concentrations (US EPA, 2002; Pronk et al., 2009; HEI, 2002); this is due in part to the fact that, in TDE, a significant fraction of DEP consists of EC (e.g. 33-90%; US EPA, 2002). However, EC is not a unique tracer for DEP in many environmental and occupational settings due to EC contributions from a variety of other common sources, including GEE, tobacco smoke, biomass smoke, and natural-gas, fuel-oil, and residual-oil combustion (HEI, 2002; Schauer, 2003). In addition, the ratio of EC to TC emissions in DE is known to vary depending on driving cycle, engine type, engine age, and engine fuel (Schauer, 2003). It is thus now well-recognized that EC measurements may not be a reliable source of exposure-response information for populations exposed to mixtures of combustion particles, such as truckers, who have historically been exposed to both DE and GEE and, frequently, tobacco smoke (HEI, 2002; Bunn et al., 2004).

Pronk et al. (2009) recently published a comprehensive review of measurement data representative of personal DE exposure levels for a variety of worker populations, including railroad workers, underground and surface mine workers, trucking company workers, bus and taxi drivers, dockworkers, construction workers, and mechanics. They included both past and current measurements (1970s up to the present) in their data compilation, although more than 80% of measurements were from the 1990s and 2000s. The larger fraction of measurements from the 1990s and 2000s illustrates one of the important limitations faced by occupational epi-demiologic studies of DE-exposed workers, namely the general lack of actual measurement data, especially for DEP, to characterize historical DE exposures.

DE exposure levels based on EC measurements from Pronk et al. (2009) are summarized in Table 2 for some DE-exposed worker populations. As shown in Table 2, there is a gradation in DE exposure levels among different DE-exposed worker populations, with the highest levels for workers in enclosed underground work sites where heavy diesel equipment has been traditionally used - e.g. in mining, mine maintenance, and construction activities. As discussed by Pronk et al. (2009), intermediate exposure levels are typical of workers using smaller equipment in above-ground (semi-) enclosed areas, such as garage mechanics, shopworkers, and dockworkers. The workers with the lowest exposure levels include truck drivers, train crew, and others who have generally worked in enclosed areas separated from DE sources. The averages in Table 2 for train crew (4-20 μg/m^3^) and for railroad maintenance workers (e.g. mechanics, shopworkers; 5-39 μg/m^3^) indicate similar exposures for these two railroad worker groups, with somewhat higher exposures on average for railroad maintenance workers. As discussed later, this is noteworthy given that a large retrospective cohort study of US railroad workers (Garshick et al., 2004; Laden et al., 2006) has reported evidence of an increased lung cancer risk among train crew, but no consistent evidence of an association between DE and lung cancer risk for railroad maintenance workers (shopworkers). For perspective, the first row of Table 2 also includes the range of average DEP levels predicted for the US states, which were modeled for 2005 year DEP emissions as part of the US EPA National-Scale Air Toxics Assessment (NATA) (US EPA, 2011).

**Table 2:** Overview of reported exposure levels for DE-exposed worker groups and the general population based on EC measurements and predicted DEP concentrations.

Population	DEP indicator	Average concentration (μg/m^3^)	Reference/comments
General population (ambient air)	DEP	0.06–2.95	US EPA (2011) – Range of modeled statewide averages for 2005 emissions inventory
Truck drivers–local	EC	2–7	Pronk et al. (2009) – Range of measured AMs from four studies
	EC	1.0 (cold), 1.2 (warm)	Davis et al. (2011) – Measured GMs for 2001–2006 TrIPS data
Truck drivers-long haul	EC	1–22	Pronk et al. (2009) – Range of measured AMs from four studies
	EC	1.1	Davis et al. (2011) – Measured GM for 2001–2006 TrIPS data
Bus drivers	EC	2–11	Pronk et al. (2009) – Range of measured AMs from four studies
Mechanics in truck terminals, bus garages, stand-alone maintenance shops	EC	4–39	Pronk et al. (2009) – Range of measured AMs from seven studies
	EC	4.3 (cold), 1.5 (warm)	Davis et al. (2011) – Measured GMs for 2001–2006 TrIPS data
Train crew	EC	4–20	Pronk et al. (2009) – Range of measured AMs from five studies
Railroad maintenance	EC	5–39	Pronk et al. (2009) – Range of measured AMs from two studies
Underground mine production workers	EC	148–637	Pronk et al. (2009) – Range of measured AMs from seven studies of various mine types (coal, metal, and non-metal)
Underground mine maintenance workers	EC	53–144	Pronk et al. (2009) – Range of measured AMs from two studies of nonmetal mines
Surface mine workers	EC	13–23	Pronk et al. (2009) – Range of measured AMs from two studies of nonmetal mines
Dockworkers	EC	4–122	Pronk et al. (2009) – Range of measured AMs from six studies
	EC	0.9	Davis et al. (2011)- Measured GM for 2001–2006 TrIPS data where propane-powered forklifts were dominant

Notes: DEP, diesel exhaust particulate; EC, elemental carbon; AM, arithmetic mean; GM, geometric mean.

EC means from Pronk et al. (2009) are for measurements using several different types of size-selective samplers (submicron, respiratory, inhalable, and not indicated). Although not shown in this table, Pronk et al. (2009) also compiled occupational exposure measurements collected using other exposure surrogates, including respirable PM, NO, NO^2^, and CO. As indicated above, Davis et al. (2011) reported separate GMs for cold- and warm-weather conditions for local truck drivers and mechanics.

**Figure 5:**
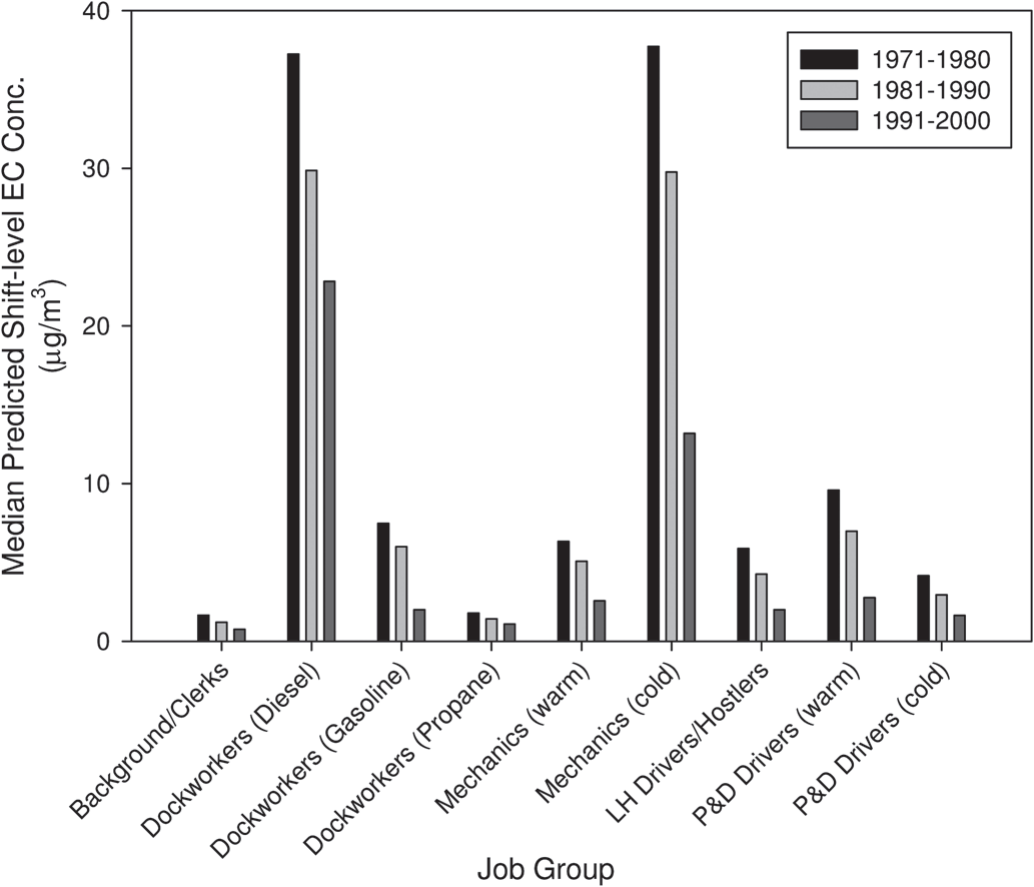
Median predicted shift-level elemental carbon (EC) concentrations for trucking industry workers by decade (1971-1980,1981-1990,1991-2000), as reported in Davis et al. (2011). Job-specific concentrations are summarized, with multiple predictions for dockworkers corresponding to use of diesel-powered, propane-powered, and gasoline-powered forklifts and separate predictions for both mechanics and pickup & delivery drivers in warm versus cold climates. As discussed in Davis et al. (2011), their modeling analysis provides evidence of substantial reductions in truckers’ DE exposures over the last three decades. LH stands for long-haul, while P&D stands for pickup-and-delivery.

Although Pronk et al. (2009) concluded that their data compilation could not be used to assess time trends in worker DE exposure levels, other recent studies provide some evidence of substantial declines in occupational and environmental DE exposure levels. In particular, Davis et al. (2011) recently published the results of their statistical modeling analysis of historical EC exposures among nationwide US trucking industry workers included in the Harvard School of Public Health retrospective epi-demiologic cohort. In constructing their model, Davis et al. (2011) combined the extensive EC measurement data collected as part of the Trucking Industry Particle Study (TrIPS, consisting of >4000 environmental samples collected between 2001 and 2006 at 36 different trucking terminals) with historical EC measurement data collected in 1988-1989 as part of the NIOSH study of Teamster unionized trucking industry workers (Zaebst et al., 1991). The study's authors developed an approach for spatial and temporal extrapolation using these data-sets and a number of assumptions. Figure 5 summarizes the model predictions of median shift-level EC concentrations for trucking workers by decade (1971-1980, 1981-1990, 1991-2000), providing evidence of marked declines in DE exposures across several classes of trucking industry workers. It is expected that these model predictions will be used in an updated epidemiologic analysis of this retrospective cohort. However, it should be noted that significant limitations have been noted in the Zaebst et al. (1991) data that are the basis for the Davis et al. (2011) temporal extrapolation approach (HEI, 1999; Bunn et al., 2004; Hesterberg et al., 2006). In particular, there is evidence suggesting that non-DE sources may have contributed significant fractions of EC and total PM exposures for the trucking workers monitored by Zaebst et al. (1991). In addition, we observe that the Zaebst et al. (1991) data were collected between 1988-1989, but Davis et al. (2011) rely upon them for modeling EC exposure concentrations back to 1971.

**Figure 6:**
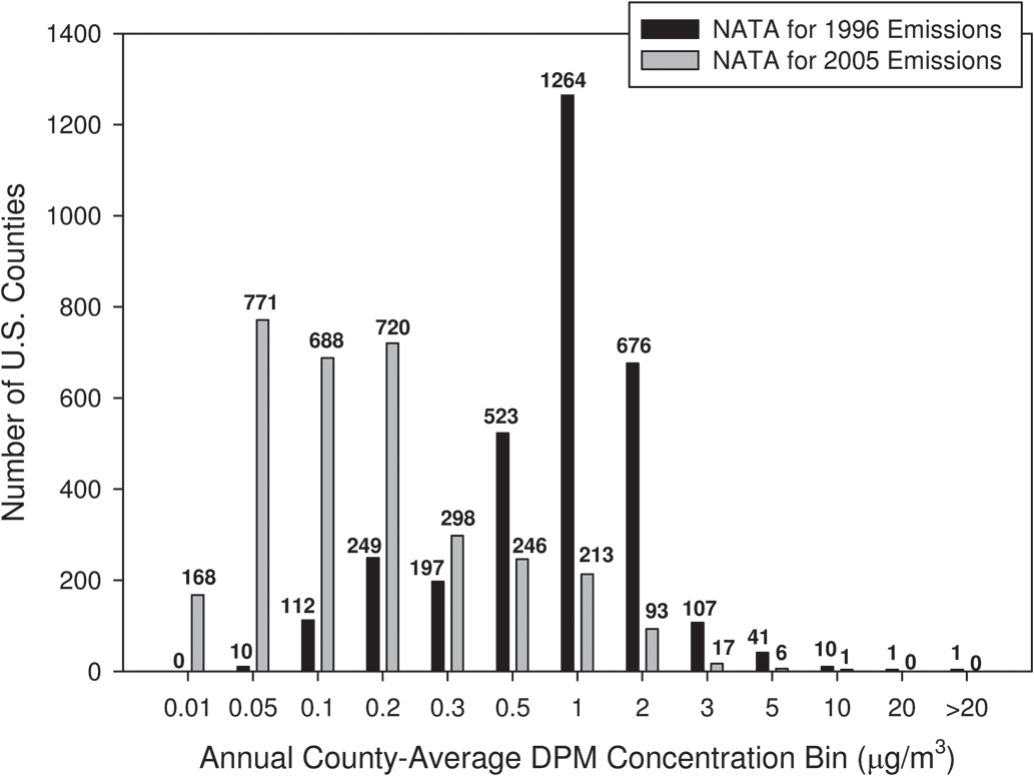
Histogram of predicted annual county-average ambient diesel particulate matter (DPM) concentrations for the US EPA National-Scale Air Toxics Assessment (NATA) modeling analyses of 1996 and 2005 year air pollutant emissions (data from US EPA, 2011). DPM emissions include both on-road and non-road emissions sources. County numbers (out of 3191 counties for the 1996 emission year modeling and 3221 counties for the 2005 emission year modeling; both including municipalities in Puerto Rico and counties in the US Virgin Islands) are provided above each bar. These data suggest a decline in ambient DE exposure levels between 1996 and 2005, although there have also been improvements in NATA methods (e.g. inventory improvements, modeling changes, background calculation revisions) over time that may affect the interpretation of any differences between the two NATA analyses.

US EPA National-Scale Air Toxics Assessment (NATA) predictions of DEP ambient air concentrations in US counties also support a downward trend in environmental DE levels (Figure 6). US EPA has now performed four NATA analyses (for 1996, 1999, 2002, and 2005 year emissions) that include air dispersion modeling of air toxics emissions from major point sources, area sources, and both on-road and non-road mobile sources (US EPA, 2011). Each NATA analysis also calculates non-cancer and cancer health risks from modeled HAP concentrations, although no cancer risks have been predicted for DEP due to US EPA's determination that the health effects data are insufficient to support the development of a cancer unit risk for DEP. Figure 6 compares predictions of county-average DEP exposure levels for the NATA analyses of 1996 and 2005 year emissions. Although it is important to acknowledge that there were changes in NATA modeling methodologies between these two analyses that could contribute to differences in their predictions, the histograms in Figure 6 show a marked shift towards lower predicted DEP concentrations for year 2005 emissions versus year 1996 emissions.

Efforts by investigators at NIOSH and the National Cancer Institute (NCI) to derive quantitative estimates of historical exposures among US underground miners further illustrate the difficulties in obtaining reliable historical exposure data for DE-exposed populations. These efforts recently culminated in the publication of five papers that detail the exposure assessment approach for the NIOSH-NCI miners study (Stewart et al., 2010; Coble et al., 2010; Vermeulen et al., 2010a, 2010b; Stewart et al., 2012). As discussed in this series of papers, these investigators selected respirable elemental carbon (REC) as the primary exposure surrogate for miners’ exposure to DEP. However, given the lack of historical REC measurements, they relied upon historical measurements of carbon monoxide (CO), as well as information on engine horsepower (HP) and mine ventilation, to “back-extrapolate” REC exposure levels to the start of diesel equipment use (1940s to 1960s, depending on the facility) from contemporaneous (1998-2001) REC exposure levels. As described in the NIOSH-NCI exposure assessment papers, CO was used as a surrogate for REC; moreover, for the lengthy period of time (1947-1976) when CO data were not available, the ratio of HP to mine ventilation rates was used as a surrogate for CO.

Even though the NIOSH-NCI investigators represent CO (and CO *via* HP) as “an optimal scientifically sound strategy” for reconstructing historical DE exposures among underground miners, it is important to note that CO has not been previously used as a surrogate to quantify DE exposure in epidemiologic studies. Based on our review of over 100 publications (i.e. papers, reports, reviews, and related exposure studies), we failed to identify a single epidemiologic analysis that relied on CO as a DE exposure surrogate. Notably, neither of the recent retrospective exposure assessments for the US railroad worker cohort (Laden et al., 2006) and the US trucking worker cohort (Davis et al., 2011) used CO data to reconstruct historical DE exposures. In fact, Davis et al. (2011) used historical data on coefficient of haze (COH), rather than CO, as a surrogate marker of EC in their retrospective assessment, explaining that COH “provides a much stronger surrogate marker of EC than does CO.” Several publications have explicitly criticized the use of CO as a DE exposure surrogate due to CO being a common combustion product that arises from many sources and is not specific to DE (Zaebst et al., 1991; Steenland et al., 1998; Verma et al., 1999).

In addition, it is well-recognized in the diesel engine industry that there is little correlation between CO and PM across engine types. Several investigators have previously examined the relationship between emissions of CO and PM for heavy-duty diesel engines, observing cases of strong CO:PM correlations for single vehicles operated within a specific cycle, but generally no consistent CO:PM relationships across different test schedules, vehicles, engines, and geographic locations (Clark et al., 1999; Jarrett and Clark, 2001; Xu et al., 2005). Based on their emissions testing of engines from several different manufacturers, Clark et al. (1999) concluded, “The wide range of average CO/PM ratios is too great to allow the inference of PM directly from CO.” More recently, McKain et al. (2012) conducted a comprehensive analysis of several large DE emissions databases, including from the E-55/59 and Gasoline/Diesel PM Split programs (see Hsu and Mullen, 2007, for a description of these datas-ets), observing weak, and highly variable, correlations between PM and CO emission rates.

Finally, the NIOSH-NCI study investigators themselves (Stewart et al., 2011), and others (Borak et al., 2011; Davis et al., 2011; Crump and Van Landingham, 2012), have highlighted the imprecision in the historical REC estimates and the potential for exposure misclas-sification. Borak et al. (2011) emphasized problems with the precision, accuracy, and reliability of the analytical methods used to historically measure CO in underground mines. In a response to the Borak et al. (2011) concerns, Stewart et al. (2011) acknowledged the imprecision in their CO measurements, and in turn, in their REC exposure estimates; however, they disagreed that this imprecision would result in false-positive findings in epidemiologic analyses, claiming instead that it was a source of nondifferential misclassification. Both Borak et al. (2011) and Davis et al. (2011) emphasized that the limited side-by-side CO and REC measurement data cited by the NIOSH-NCI investigators support only a relatively modest correlation between CO and REC (r = 0.4, i.e. r^2^ = 0.16; Vermeulen et al., 2010a). Based on the lack of strong correlation, Davis et al. (2011) concluded that, “the use of CO as a surrogate for EC may lead to exposure misclassification bias.” Crump and Van Landingham (2012) outlined the uncertainties in each step of the NIOSH-NCI exposure assessment, in particular demonstrating the lack of support for the NIOSH-NCI assumption of a linear relationship between CO and REC and for the NIOSH-NCI assumed relationship between HP and CO. In attempting to reconstruct the NIOSH-NCI exposure assessment and propagate uncertainties through the various steps, they demonstrated how moderate changes intended to improve the NIOSH-NCI methodology had significant impacts on the resulting exposure estimates. Large differences were observed between 5th and 95th percentiles for REC historical predictions, even without accounting for the full uncertainty of the REC exposures (e.g. there was no consideration of uncertainties related to data on engine horsepower and the rate of mine air exhaust, due to the lack of available information). Based on the Crump and Van Landingham (2012) graphical comparisons of the two sets of REC historical predictions for mine operators, median NIOSH-NCI estimates are frequently larger than the re-constructed median REC estimates for most, but not all, mines.

Finally, given no mention of DOCs in the NIOSH-NCI exposure assessment papers, it is unclear how the NIOSH-NCI investigators accounted for the 1970s introduction of the DOC as an aftertreatment technology for diesel-powered mining equipment (DieselNet, 2004). DOCs gained usage at many underground mines in the 1970s and 1980s due to their ability to efficiently convert CO in the exhaust stream to CO_2_ (DieselNet, 2004), meaning that their use greatly decreased ratios of CO/REC and, in effect, reduced the direct linkage between CO and REC. Thus, even if one accepted the assumption that in some mines CO correlated well with REC, which appears to not be true, there can be no correlation between CO and REC at mines employing DOCs.

### Concluding remarks on DE exposure assessment

Paired with emissions data for newer diesel engines, DE exposure measurements provide further support for the changing nature of DE exposures, specifically for a decrease in DE exposure levels over time. Despite increases in the use of diesel engines over the last several decades, there is evidence that emissions reductions are contributing to reduced occupational and environmental DE exposure levels. Overall, however, DE exposure assessment remains an inexact science both for current and for historical DE exposures. Recent efforts have employed predictive time-trend models to attempt to reconstruct quantitative estimates of historical DE exposures, but these modeling approaches are recognized to yield imprecise and uncertain exposure estimates due to their reliance on numerous assumptions and uncertain data. It is clear that additional efforts are needed to continue to study the role of NTDE on occupational and environmental DE exposure levels, especially as traditional diesel engines are replaced with new technology diesel engines. Given the significant reductions in EC emissions, this may necessitate the development of additional surrogates of DE exposure.

## State of the knowledge regarding DE carcinogenic potential

As documented in Table 3, we now have more than five decades of DE health effects research. Table 3 focuses in particular on the research addressing DE carcinogenic potential, providing a roadmap of the intensive experimental and epidemiologic research efforts that followed US EPAs 1977 announcement of preliminary findings of mutagenicity in bacterial assays of organic solvent extracts of DEP, and US EPAs decision to launch a major health effects research program (NY Times, 1977; Huisingh et al., 1978). In the late 1970s, there were only a handful of epidemiologic studies of DE-exposed workers (e.g. Hueper, 1956; Raffle, 1957; Kaplan, 1959), but about 10 years later, IARC (1989) reviewed approximately 20 epidemiologic studies of DE-exposed workers as bearing on the relationship between DE exposure and lung cancer risk. By this time, findings were available for 6 of the 10 large-scale (50 or more animals per group) chronic inhalation DE tumorigenicity bioassays that had been conducted in rats. Since the 1988 IARC assessment (IARC, 1989), several additional large-scale chronic inhalation DE rat bioassays have been conducted (but only one since 2000, namely Stinn et al., 2005), and there has been a steady trickle of epidemiologic analyses of lung cancer risk among DE-exposed workers. In the last decade, researchers have noted a shift in DE health effects research from its heavy focus on lung cancer risk to a broadened focus on both potential non-cancer and cancer health hazards (Mauderly and Garshick, 2009). This shift approximately coincided with the introduction of the PM_2.5_ indicator for the PM NAAQS.

**Table 3:** Timeline of key DE health effects research milestones.

Year	Event
1955	Kotin et al. (1954, 1955) publish first evidence of carcinogenicity of DE soot extracts based on mouse skin assay
Mid- to late 1950s	Hueper (1956), Raffle (1957), and Kaplan (1959) publish earliest epidemiologic analyses of lung cancer rates among railroad workers with diesel exhaust exposure, reporting conflicting findings
1978	Using bacterial assays (Ames Test), Huisingh et al. (1978) report first evidence of mutagenicity of organic extracts of DE soot
1979	1st US EPA international symposium on the health effects of diesel engine emissions held in December in Cincinnati, OH
1980	Health Effects Institute (HEI) formed as a nonprofit organization to help develop a database on the health effects of pollutants from motor vehicles and other environmental sources
1981	National Research Council (NRC) releases report “Health Effects of Exposure to Diesel Exhaust,” authored by the Health Effects Panel of NRC's Diesel Impacts Study Committee; 2 US EPA diesel emissions symposium held in October in Raleigh, NC
1982	Symposium on Biological Tests in the Evaluation of Mutagenicity and Carcinogenicity of Air Pollutants with Special Reference to Motor Exhausts and Coal Combustion Products held in February in Stockholm, Sweden
Early 1980s	Flurry of studies (e.g. Brooks et al., 1984; Clark et al., 1981, 1984; Claxton, 1981, 1983; Lewtas, 1982, 1983; Siak et al., 1981) confirm mutagenicity of DPM extracts in bacterial and mammalian cells assays, showing large variability in mutagenic potency of soot extracts depending on such factors as engine, fuel type, operating conditions; early focus on potential health impacts of organic chemical constituents of DE
Early to mid-1980s	Early retrospective mortality cohort studies of occupational DE exposures and lung cancer (e.g. Waller, 1981; Howe et al., 1983; Rushton et al., 1983; Wong et al., 1985; Gustafsson et al., 1986)
1983	Zamora et al. (1983) report evidence that components of diesel extract act as weak tumor promoters
1986	International Satellite Symposium on Toxicological Effects of Emissions from Diesel Engines held in July in Tsukuba Science City, Japan
1986–1987	Early development of lung overload concept – Vostal (1986) proposes hypothesis that lung tumor development in rats exposed to highly elevated DE concentrations is due to consequences of lung overload in rats; Wolff et al. (1987) publish key paper developing concept of lung overload
Mid- to late 1980s	Initial series of findings from large-scale (50 or more animals per group) chronic inhalation DE carcinogenicity bioas-says (e.g. Heinrich et al., 1986; Mauderly et al., 1986, 1987; Takemoto et al., 1986; Ishihara, 1988; Brightwell et al., 1989; Lewis et al., 1989); additional epidemiological studies published, including early analyses of US railroad workers (Garshick et al., 1987, 1988) and large general population cohorts (Boffetta et al., 1988; Boffetta and Stellman, 1988)
Early 1990s	US EPA releases first draft of its “Health Assessment Document for Diesel Engine Exhaust”; first studies of DE expo-sures and lung cancer risks of Teamsters Union trucking industry workers published (Steenland et al., 1990, 1992; Zaebst et al., 1991)
1995	Health Effects Institute's (HEI's) Diesel Working Group releases special report “Diesel Exhaust: A Critical Analysis of Emissions, Exposure, and Health Effects,” which includes critical review of all published epidemiologic stud-ies available through June 1993 bearing on the lung cancer risk posed by occupational DE exposure (35 in total); Massachusetts Institute of Technology (MIT) Toxicology Symposium “Particle Overload in the Rat Lung and Lung Cancer: Relevance for Human Risk Assessment” held in Cambridge, MA
1995–1996	Second wave of published findings for chronic inhalation carcinogenicity bioassays with groups of 50 or more ani-mals (e.g. Heinrich et al., 1995; Nikula et al., 1995; Mauderly et al., 1996)
Mid- to late 1990s	Emerging consensus that findings of rat lung tumors at highly-elevated DE exposure levels are due to non-specific response to a high lung burden of particles (i.e. lung overload) rather than response to specific DE mutagens (e.g. PAHs, nitro-PAHs), and that rat findings may be of little relevance to human lung cancer risk from environmental DE exposures (Oberdorster, 1995; Mauderly, 1996, 1997, 2000; Mauderly and McCunney, 1996; Valberg and Crouch, 1999; ILSI, 2000; US EPA, 2002)
Mid-1990s to mid-2000s	Burgeoning number of literature reviews addressing the health effects evidence for DE and lung cancer risk, including Mauderly (1994), HEI (1995), Muscat and Wynder (1995), IPCS (1996), Stober and Abel (1996), Morgan et al. (1997), Stober et al. (1998), Lloyd and Cackette (2001), US EPA (2002); Bunn et al. (2004), Hesterberg et al. (2005, 2006)
1997–1999	Early meta-analyses (e.g. Bhatia et al., 1998; Lipsett and Campleman, 1999) and re-analyses of epidemiologic data (e.g. Cox 1997; Cal EPA, 1998; Crump, 1999) bearing on occupational DE exposure and lung cancer risk
1998	Updated Teamsters Union epidemiological study published (Steenland et al., 1998)
1999	HEI's Diesel Epidemiology Expert Panel releases report “Diesel Emissions and Lung Cancer: Epidemiology and Quantitative Risk Assessment” that concludes that reliable epidemiologic data are not currently available to support a quantitative risk assessment for DE exposure and lung cancer risk; meta-analysis of chronic inhalation carcinoge-nicity bioassay data published by Valberg and Crouch (1999)
Late 1990s-present	Periodic publication of additional cohort (Saverin et al., 1999; Jarvholm and Silverman, 2003; Neumeyer-Gromen et al., 2009) and population-based case–control epidemiological studies of occupational DE exposure and lung cancer risk (e.g. Bruske-Hohlfeld et al., 1999; Gustavsson et al., 2000; Boffetta et al., 2001; Soll-Iohanning et al., 2003; Guo et al., 2004; Richiardi et al., 2006; Parent et al., 2007; Villeneuve et al., 2011); Increasing number of ambient air pollu-tion epidemiological studies reporting associations between exposure to traffic-related air pollution and adverse health outcomes, including asthma exacerbation, cardiovascular and respiratory morbidity, and premature mortality (recently reviewed in HEI, 2010)
~2000	Approximate time-period of shift in DE health effects research from predominant focus on lung cancer risk to broad range of potential non-cancer health hazards (Mauderly and Garshick, 2009)
2002	US EPA releases final “Health Assessment Document for Diesel Engine Exhaust”; HEI Diesel Epidemiology Working Group releases special report “Research Directions to Improve Estimates of Human Exposure and Risk from Diesel Exhaust” that recommended a series of short-term, medium-term, and long-term research activities intended to enhance the epidemiologic evidence addressing disease risks associated with diesel emissions
2003	Workshop, jointly organized by HEI and CRC, held in Denver, Colorado, to begin the process of developing an approach and guidelines for ACES emissions characterization and health effects evaluation
2004–2006	Updates published for US railroad worker cohort with increased years of follow-up and refinements to models and exposure assessment (e.g. Garshick et al., 2004, 2006; Lee et al., 2004; Laden et al., 2006)
2005	Hesterberg et al. (2005) propose the term “New Technology Diesel Exhaust (NTDE)” to differentiate the exhaust from post-2006 advanced diesel engines with integrated, multi-component emissions reduction systems (modern electronic fuel injection systems, ultra-low-sulfur fuel, special lubricants, and exhaust aftertreatment devices such as diesel particulate filters) with DE from pre-2006 diesel engines; Stinn et al. (2005) publish most recent chronic inhalation carcinogenicity bioassay with groups of 50 or more animals
Mid-2000s to present	Series of exposure assessment studies (Lee et al., 2005; Smith et al., 2006; Davis et al., 2006, 2007a, 2007b, 2011; Sheesley et al., 2008, 2009) and epidemiologic studies (Laden et al., 2007; Garshick et al., 2008) published by researchers at the Harvard School of Public Health as part of National Cancer Institute funded cohort study of lung cancer in the US trucking industry
2007	Beginning of ACES emissions and toxicological testing of exhaust from new technology diesel engines meeting the 2007/2010 emissions standards
2010	Series of studies published detailing the estimation of historical DE exposures among workers at underground non-metal mining facilities (Stewart et al., 2010; Coble et al., 2010; Vermeulen et al., 2010a, 2010b)
2010–2011	Recent critical reviews and re-analyses of epidemiological data for historical occupational DE exposure and lung cancer risk (e.g. Gamble, 2010; Olsson et al., 2011a)
2012	Epidemiology papers published for NIOSH-NCI Diesel Exhaust in Miners Study (DEMS), including a cohort mortality study (Attfield et al., 2012) and a nested case-control study of lung cancer mortality (Silverman et al., 2012), each conducted for DE-exposed miners at eight US non-metal mining facilities
2013	Final reports expected detailing the findings of the ACES chronic inhalation bioassays for exhaust from new technology diesel engines meeting the 2007/2010 emissions standards

Over time, major reviews of the DE health effects research have been prepared (e.g. NRC 1981; McClellan, 1987; NIOSH, 1988; IARC, 1989; HEI, 1995; Muscat and Wynder, 1995; Stober and Abel, 1996; IPCS, 1996; Cox, 1997; Morgan et al., 1997; Cal EPA, 1998; Lloyd and Cackette, 2001; US EPA, 2002; IOM, 2005; Hesterberg et al., 2005, 2006, 2009, 2011; Mauderly and Garshick, 2009; Gamble, 2010; Gamble et al., 2012), as well as a variety of focused critical assessments and commentaries (e.g. McClellan, 1986; Silverman, 1998; Stober et al., 1998; HEI, 1999, 2002; Bunn et al., 2004; Rogers and Davies, 2005; Wichmann, 2007; Ward et al., 2010; Laumbach and Kipen, 2011). Given the availability of recent in-depth reviews by leading health effects researchers and authoritative bodies, we do not provide an exhaustive evaluation of either the epidemiologic or experimental evidence bearing on DE carcinogenic potential. Instead, we examine major advances in epidemiologic and experimental evidence since the last IARC evaluation, focusing in particular on recent, notable pieces of scientific evidence. As we emphasize below, even now, most research studies and major hazard assessments bearing on DE carcinogenic potential are relevant to TDE and not NTDE. This is because exposures in these studies are nearly exclusively to DE from pre-2006 engines, and most commonly, pre-1988 engines (i.e. pre-regulation of DEP emissions). In the final parts of this section, we briefly touch upon the current thinking regarding the carcinogenic potential of NTDE, and draw comparisons between the particulate emissions in NTDE with those in contemporary GEE.

### Human epidemiology of TDE

A number of health effects researchers have weighed the epidemiologic evidence relevant to DE exposure and lung cancer over the years, with some (Bhatia et al., 1998; Lipsett and Campleman, 1999; Lloyd and Cackette, 2001; US EPA, 2002; Wichmann, 2007) concluding that there is sufficient evidence to support a causal role for DE in lung cancer risk. As discussed later, several regulatory agencies and authoritative bodies have concluded that DE is a “likely,” “reasonably anticipated,” or “probable” carcinogenic hazard (e.g. IARC, 1989; HEI, 1995; IPCS, 1996; Cal EPA, 1998; NTP, 2000; US EPA, 2002). Other reviewers (Stober and Abel, 1996; Muscat and Wynder, 1995; Cox, 1997; Morgan et al., 1997; Bunn et al., 2004; Hesterberg et al., 2005, 2006; Gamble, 2010) have instead concluded that inconsistencies and limitations in the available data prevent making a causal link to lung cancer. Scientists on both sides of the question have highlighted a variety of deficiencies and uncertainties in the body of DE epidemiologic findings that now includes more than 50 published occupational cohort and case-control studies.

In its 1988 assessment that reviewed approximately 20 epidemiologic studies, IARC (1989) noted several issues, including a general lack of quantitative data on workers’ DE exposures, a reliance on job/industry titles for inferring group-level exposures, inadequate control of smoking and other potential confounders (e.g. asbestos, radon, lifestyle), and difficulties in separating out risks due to DE versus other engine exhausts. In 1995, the Health Effects Institute (HEI, 1995) reviewed an expanded set of over 30 epidemiologic studies, reaching a similar determination regarding the notable limitations in the available studies. In particular, HEI concluded that “the lack of definitive [DE] exposure data for the occupationally exposed study populations precludes using the available epidemiologic data to develop quantitative estimates of cancer risk.” In its 2002 Health Assessment Document for Diesel Engine Exhaust (hereafter referred to as the Diesel HAD), US EPA (2002) focused their analysis on 22 epidemiologic studies, concluding that the interpretation of the epidemiologic findings was complicated by such factors as the lack of “actual” DE exposure data, the role of potential confounders, and the lack of evidence for an exposure-response relationship. More recently, Gamble (2010) concluded that several limitations and uncertainties (e.g. inadequate latency, a random pattern of small increased lung cancer risks, exposure misclassification, impacts of potential confounders such as cigarette smoke and pre-diesel era exposures, and inconsistent evidence of positive exposure-response trends) continue to cloud the interpretation of the DE epidemiologic evidence.

Table 4 summarizes study design characteristics, key findings, and notable limitations of the 19 epidemiologic studies published over the last decade (i.e. post-US EPA HAD) that we identified as being important to evaluating the DE-lung cancer link. As shown in this table, many of these studies have strengths in their design, including large sample sizes, semi-quantitative (e.g. based on an expert job-exposure matrix [IEM]) and sometimes quantitative exposure assessments, reasonable data on smoking, and control of other potential occupational carcinogens (e.g. GEE, silica, asbestos). However, Table 4 also shows that, in general, these recent studies are still hindered by notable limitations, including inadequate latency, incomplete adjustment for smoking, no measured historical DE exposure data, and unmeasured confounding variables (e.g. pre-diesel era exposures, non-diesel PM exposures, other job category differences). Table 4 shows the predominant working periods for study subjects, providing evidence of the mixed exposures received by many workers that included significant exposures during pre-diesel years. In addition, it shows that any diesel exposures were likely dominated by emissions from pre-1988 (i.e. pre-regulation of DEP emissions) diesel engines. Below, we examine the question as to whether these recent studies have addressed some of the well-recognized limitations and strengthened the body of epidemiologic evidence. We focus in particular on the latest findings for those worker cohorts considered to offer the most informative datasets for examining the DE-lung cancer relationship, namely railroad workers, trucking industry workers, and underground miners. We also highlight two recent case-control studies (Olsson et al., 2011a; Villeneuve et al., 2011) distinguished from prior studies by large numbers of cases and more refined exposure assessments. Additional analyses of the strengths and limitations of most of these recent studies are available in comprehensive reviews prepared by Mauderly and Garshick (2009), Gamble (2010), Gamble et al. (2012), and IOM (2005).

**Table 4:**
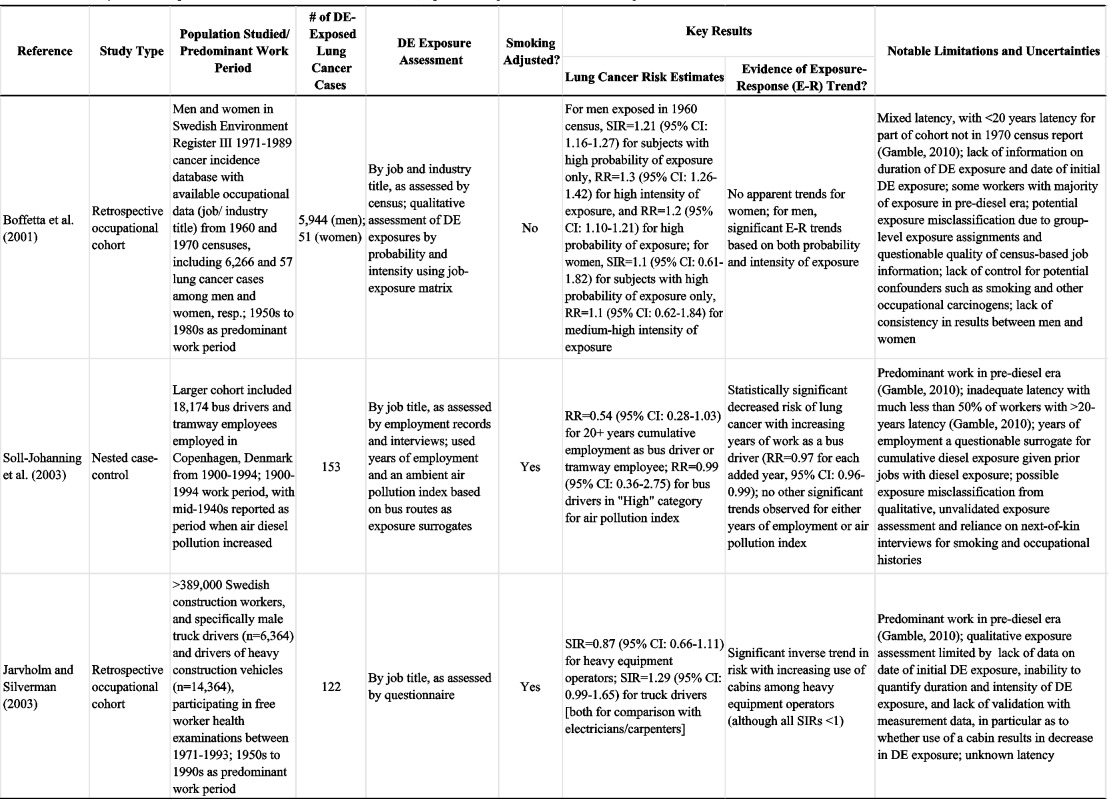

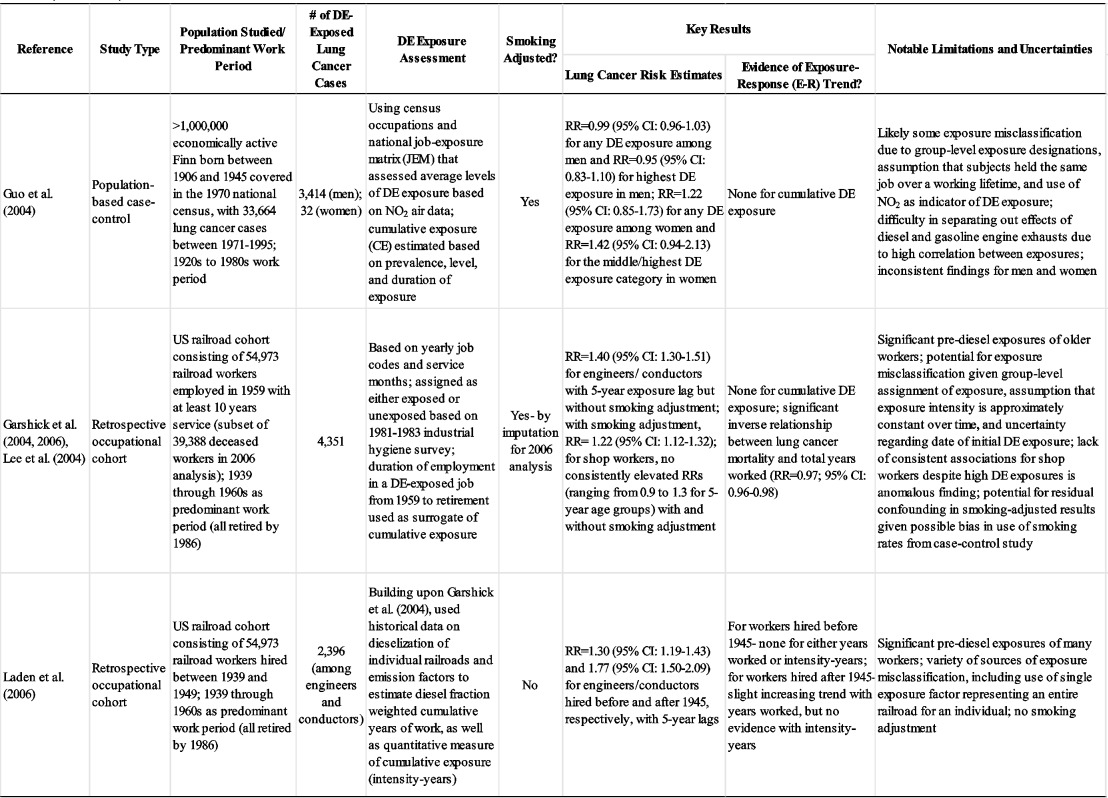

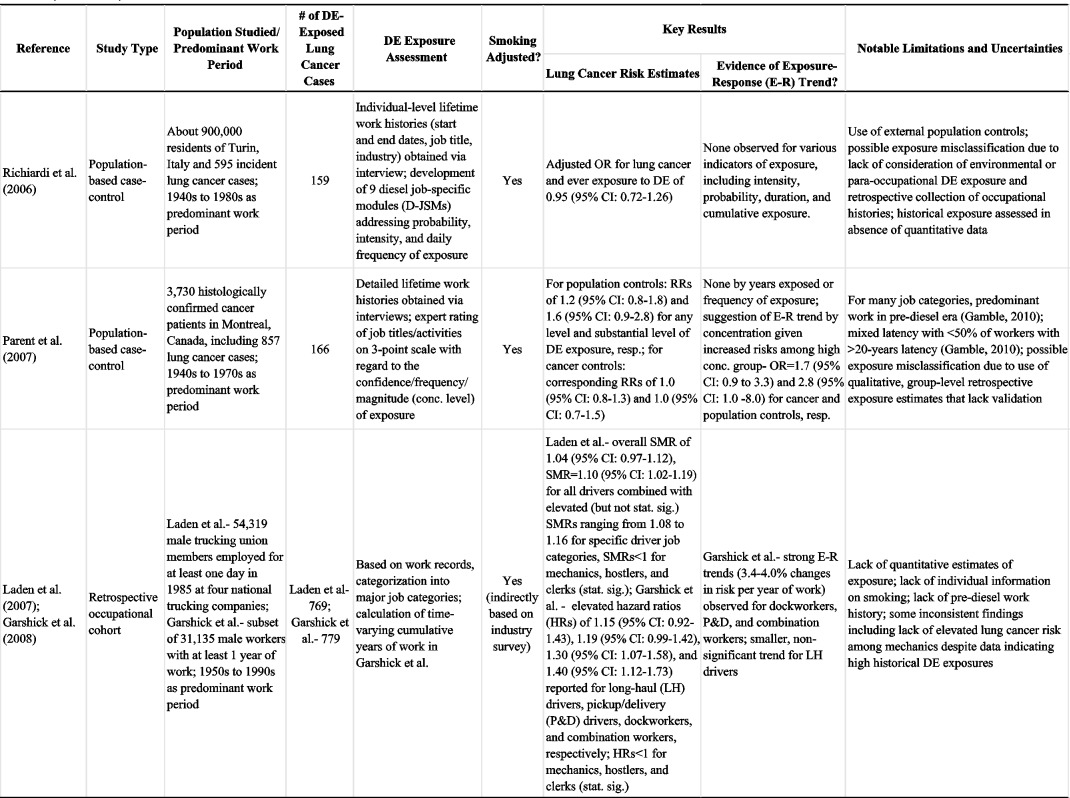

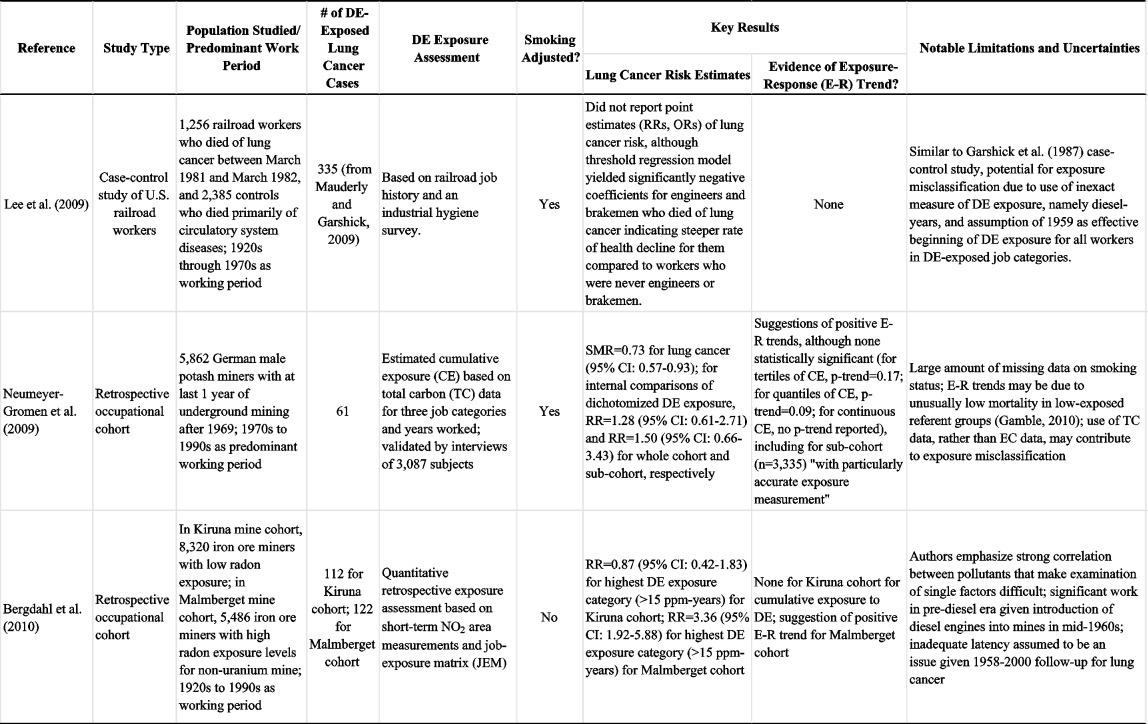

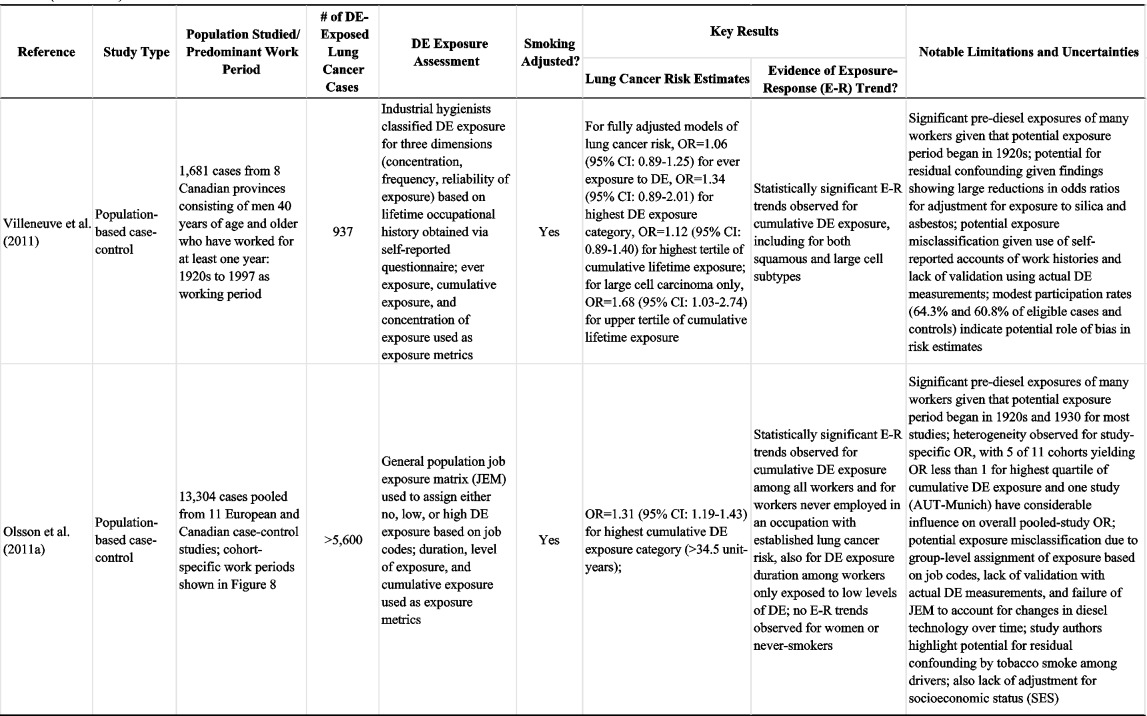

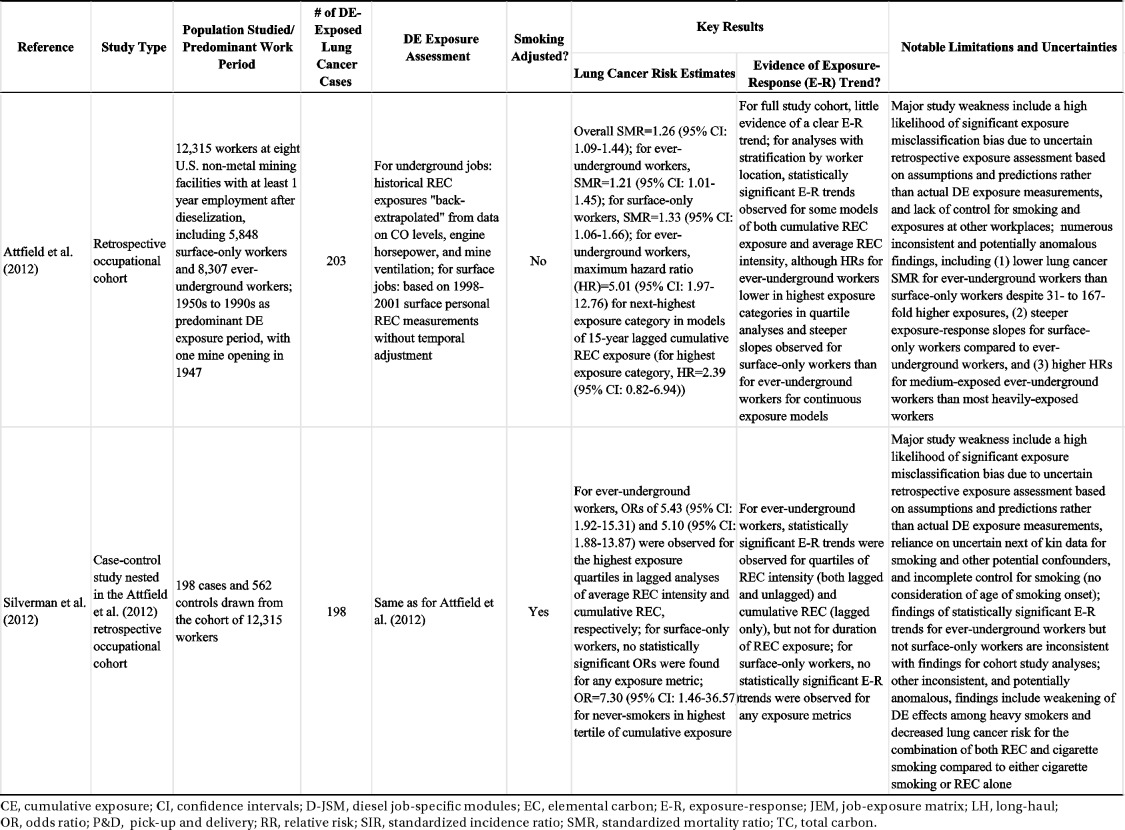
Summary of recent (post-US EPA Diesel HAD, 2001 and later) epidemiological studies of DE-lung cancer risk.

As summarized in Table 4, updated analyses were published in 2004 (Garshick et al., 2004) and 2006 (Garshick et al., 2006; Laden et al., 2006) for the large retrospective cohort of US railroad workers (>50,000 former workers) that was originally analyzed by Garshick et al. (1988). Shortly after IARC's 1988 assessment that cited Garshick et al. (1988) as a key epidemiologic study, Crump et al. (1991) conducted a detailed re-analysis of the Garshick et al. (1988) data, identifying some methodological problems and inconsistent results. These included evidence of incomplete follow-up, as well as a lack of increased lung cancer risks for shopworkers, despite exposure measurements indicating that these workers had the highest DE exposures (see Crump, 1999, 2001, as well as Hesterberg et al., 2006; Gamble, 2010). In addition, based on analyses using data with complete follow-up, more careful correction of age, and accurate quantification of years of exposure, Crump (1999, 2001) demonstrated a negative exposure-response trend for train crew workers (i.e. reduced lung cancer risk with increased duration of exposure, as well as for quantitative measures of cumulative exposure).

The 2004 and 2006 updated cohort analyses corrected the incomplete follow-up identified by Crump, extended cohort follow-up by an additional 16 years (now covering the period 1959 to 1996), examined the potential confounding role of smoking, refined the DE exposure assessment, and further investigated exposure-response trends. Notably, Laden et al. (2006) developed an innovative quantitative metric of estimated cumulative exposure, called “intensity-years” which factored in annual railroad-specific weighting factors for the probability of diesel exposure, as well as train-specific emissions factors, to estimate average annual exposure intensities. Similar to the original study findings, these updated analyses reported elevated lung cancer mortality risks for train crew workers, including relative risks (RRs) of 1.30 (95% CI: 1.19-1.43) and 1.77 (95% CI: 1.50-2.09) for engineers/conductors hired before and after 1945, respectively, for analyses with 5-year lags (Laden et al., 2006). However, Garshick et al. (2004) again did not observe any consistent increase in lung cancer risks for shopworkers (note that Laden et al. do not provide specific results for shopworkers).

These updated analyses also yielded inconsistent findings related to DE exposure-response trends, supporting the concerns raised by Crump (1999, 2001). In agreement with the Crump (1999, 2001) findings, Garshick et al. (2004) observed no increase in risks with increasing years of work (their exposure surrogate for cumulative exposure) in an engineer or conductor job, and further, a statistically significant decrease in lung cancer mortality with total years worked (RR = 0.97; 95% CI: 0.96-0.98). Laden et al. (2006) noted an “apparent exposure response” with increasing cumulative years of work for their analyses of workers hired after 1945 when the introduction of diesel locomotives approximately began, but not for analyses of workers hired before 1945. Laden et al. (2006) observed no evidence of an exposure-response trend with their refined “intensity-years” exposure metric for cumulative exposure. Garshick et al. (2004) acknowledged a potential confounding role of pre-diesel era exposures in their findings given that it was not until 1959 when the transition from coal-powered to diesel-powered locomotives was 95% complete in the US (Garshick et al., 1988; Gamble, 2010).

Laden et al. (2007) and Garshick et al. (2008) also pub -lished analyses of lung cancer risks among a large cohort of US trucking workers (>54,000 men), with follow-up from 1985 to 2000. Earlier case-control analyses of lung cancer risk among US trucking workers (Steenland et al., 1990, 1992, 1998) reported evidence of positive exposure-response trends, but had a variety of limitations that included inadequate latency, possible misclassification of smoking habits due to use of next-of-kin (NOK) data, and uncertain exposure estimates that were based on “broad assumptions rather than actual measurements.” As summarized in Table 4, the Garshick et al. (2008) re-analysis also lacked actual measures of historical DE exposure, relying instead on work records to categorize workers into major job categories and to estimate cumulative years of work. The Laden et al. (2007) and Garshick et al. (2008) results are supportive of elevated lung cancer risks among truck drivers and dockworkers, but not mechanics, hostlers, and clerks. Table 4 shows standardized mortality ratios [SMRs] for lung cancer reported for the Laden et al. analysis, which used the general US population as the comparison population, and lung cancer hazard ratios (HRs) reported for the Garshick et al. proportional hazard regression analysis, which used internal cohort-based reference groups. In addition, Garshick et al. (2008) reported strong evidence of exposure duration-response trends, including statistically significant 3.4 to 4.0% changes in lung cancer risk per year of work for dockworkers, pickup/delivery (P&D) drivers, and combination workers, and a smaller, non-significant trend for long-haul (LH) drivers.

As noted by others (HEI, 1999; Hesterberg et al., 2006; Gamble, 2010), the attribution of a specific role of DE exposure to the observed lung cancer increases among some types of trucking industry workers is hindered by both the lack of quantitative historical DE exposure data, as well as the lack of quantitative data characterizing historical GEE exposures. Other reviews (Hesterberg et al., 2006; Gamble, 2010) have also discussed the sizeable number of epidemiologic studies reporting increased lung cancer risks among pre-diesel era drivers and the lack of any apparent change in lung cancer risks after truck dieselization, both of which provide support for the hypothesis that another work-related exposure or a lifestyle factor may underlie the increased lung cancer risks among some trucking workers. Garshick et al. (2008) recognized difficulties in interpretation and the fact that DE is but one of many potential exposures in the trucking industry, concluding, “Trucking industry workers who have had regular exposure to vehicle exhaust from diesel and other types of vehicles on highways, city streets, and loading docks have an elevated risk of lung cancer with increasing years of work.” As noted earlier, this team of investigators recently completed a new retrospective exposure assessment of DE exposures (Davis et al., 2011) and plan to conduct updated epidemiologic analyses using the quantitative predictions of historical DE exposures (Ward et al., 2010). It is assumed that future analyses will further address what appears to be an inconsistent finding in the current epidemiologic analyses, namely the lack of increased cancer risks among mechanics in the face of exposure data indicating that they have historically been among the more heavily DE-exposed worker groups.

Compared to railroad workers and truckers, DE-exposed underground miners have been less extensively studied in relation to their lung cancer risk. There are a number of epidemiologic studies of various mining populations, however, very few have been conducted to specifically examine the relationship between DE exposure and lung cancer. This is the case despite a prevailing belief that epidemiologic study of underground miners may be particularly informative for examining the DE-lung cancer question, providing that their historically elevated DE exposures can be reliably estimated (Hesterberg et al., 2006; Silverman, 1998). Notable advantages of underground miners compared to other occupational cohorts include a history of markedly elevated DE exposure levels (e.g. 1-2 orders of magnitude higher than those of railroad workers and trucking workers; see Table 2), sufficient latency, and less potential confounding by GEE and other ambient combustion products. However, one notable disadvantage involves potential confounding from a suite of other carcinogens, including radiation (radon), asbestos, crystalline silica, and metals such as arsenic.

Initiated approximately 20 years ago, the recently published NIOSH-NCI epidemiologic analyses (Attfield et al., 2012; Silverman et al., 2012) provide some of the strongest findings to date supporting a DE-lung cancer relationship among miner populations. However, as discussed below and summarized in Table 4, the Diesel Exhaust in Miners Study (DEMS) also has its own set of limitations and uncertainties, as well as a number of inconsistent findings, that raise questions regarding the interpretation of the DEMS findings. In particular, the NIOSH-NCI exposure assessment methodology, described previously as “back-extrapolating” historical estimates of REC from CO measurement data and information on engine HP and mine ventilation, is the foundation for both epidemiologic studies and the source of estimates of cumulative REC exposures and average intensity REC exposures. As discussed below, Attfield et al. (2012) reported lung cancer SMRs for external analyses using state-based mortality rates and lung cancer HRs for Cox proportional hazard regression analyses for the full DEMS cohort of 12,315 workers from eight US non-metal mining facilities (one limestone, three potash, one salt, and three trona mines). Silverman et al. (2012) reported lung cancer ORs for a nested-case control study of the full cohort that focused on 198 lung cancer deaths and 562 incidence density-sampled control subjects. Below and in Table 4, we discuss only a subset of the findings from these two studies (that together total over 400 statistical comparisons); we direct the reader to the Attfield et al. (2012) and Silverman et al. (2012) papers for a complete picture of the DEMS epidemiologic analyses and findings.

As summarized in Table 4, Attfield et al. (2012) reported an elevated lung cancer SMR for the complete DEMS cohort (SMR = 1.26, 95% CI: 1.09-1.44). However, for separate analyses stratified by worker location (i.e. surface or underground), they observed a higher SMR for surface-only workers (SMR = 1.33, 95% CI: 1.06-1.66) than for ever-underground workers (SMR = 1.21, 95% CI: 1.01-1.45), despite ever-underground workers having 31-to 167-fold higher mean REC exposure levels. For their Cox proportional hazard regression analyses, Attfield et al. (2012) stated that, “Initial analyses from the complete cohort did not reveal a clear relationship of lung cancer mortality with DE exposure.” It was only with stratification by worker location that some evidence of statistically significant elevated HRs and positive exposure-response trends was observed. In particular, for analyses of ever-underground workers with 5 or more years of tenure and 15-year lagged cumulative REC exposures, Attfield et al. (2012) observed a maximum HR of 5.01 (95% CI: 1.97-12.76) for the next-highest exposure category (640 to <1280 μg/m^3^-y category); however, they observed a twofold lower HR of 2.39 (95% CI: 0.82-6.94) for the highest exposure category (>1280 μg/m^3^-y category), with other findings also providing evidence of a “plateauing” of risk at higher levels of REC exposure.

Attfield et al. (2012) tested for exposure-response trends using a variety of models of ever-underground workers, surface-only workers, and the complete cohort adjusted for worker location. For continuous log-linear models that considered the full range of 15-year lagged cumulative REC exposures or average intensity REC exposures, statistically significant exposure-response trends were observed for the complete cohort adjusted for worker location, but not for ever-underground workers (both restricted to workers with 5 or more years of tenure). For ever-underground workers, statistically significant exposure-response trends were, however, observed for continuous log-linear models where cumulative REC exposures were limited to less than 1280 μg/m^3^-y and for models of log continuous exposures (for both cumulative REC and average REC intensity). Despite significantly lower REC exposures, greater exposure-response coefficients were estimated for surface-only workers for both average REC intensity (HR = 2.60 versus 1.26 per log μg/m^3^; statistically significant difference) and cumulative REC exposure variables (HR = 1.02 versus 1.001 per μg/m^3^-y; statistically non-significant difference).

In contrast to the Attfield et al. (2012) cohort analyses, the Silverman et al. (2012) nested-case control study sought to control for potential confounders such as smoking and other employment in high-risk occupations for lung cancer based on information obtained from NOK interviews. For models of the combined dataset of underground and surface workers, Silverman et al. (2012) reported statistically significant or borderline statistically significant positive exposure-response trends for each of the three exposure variables they considered, namely cumulative REC exposure (lagged and unlagged analyses), average intensity REC exposure (lagged and unlagged analyses), and duration REC exposure (unlagged analyses only; apparently, no lagged analyses were conducted). For analyses stratified by work location, however, Silverman et al. (2012) reported statistically significant positive exposure-response trends for ever-underground workers, but not for surface-only workers (Table 4).

Recognizing the significant differences in the exposure-response analyses between the two studies (e.g. the cohort study modeled exposure as a continuous variable, while the nested case-control study modeled exposure as a categorical variable; the nested case-control study was able to control for additional confounders such as smoking and other occupational exposures), these findings thus differ from those for the Attfield et al. (2012) cohort analyses where steeper exposure-response slopes were observed for surface-only workers than ever-underground workers. Silverman et al. (2012) also highlight examples of higher lung cancer risk estimates for their case-control analyses of underground workers than for the Attfield et al. (2012) cohort analyses. They hypothesize that these differences may in part be due to potential negative confounding effects from cigarette smoking; in support of this hypothesis, they highlight evidence of an inverse relationship between smoking status and DE exposure in underground workers (i.e. 36% and 21% current smokers in the lowest and highest cumulative REC tertiles, respectively).

Silverman et al. (2012) also conducted analyses to examine the combined effect of diesel exposure and intensity of cigarette smoking. For analyses where nonsmoking cases (14 of 198 cases) and controls (178 of 562 controls) were categorized according to tertiles of cumulative REC exposure (lagged 15-y), an OR of 7.3 (CI = 1.46 to 36.57) was observed for the highest exposure tertile. They reported evidence of a positive exposure-response trend with cumulative REC exposure (lagged 15-y) among nonsmokers and workers who smoked less than two packs per day, but not for heavier smokers (two packs per day or greater) where ORs were observed to decrease with greater cumulative REC exposure. Similarly, when comparing workers in the lowest tertile of cumulative REC exposure, heavier smokers (two packs per day or greater) had lung cancer risks 27 times higher than non-smokers, but their lung cancer risks were only 2.5-times higher than nonsmokers for workers in the highest tertile of cumulative REC exposure.

The DEMS epidemiologic studies have some notable advantages compared to prior epidemiologic studies of miners and other worker populations, including the large cohort size, lengthy follow-up and hence adequate latency, highly elevated DE exposures, control for smoking and other workplace exposure to carcinogens (nested case-control study only), and low exposures to other potential mining-related exposure confounders including silica, asbestos, radon, and respirable dust (as supported by contemporaneous measurement data only; as for REC, historical exposure data are also absent for other mining-related exposures). Although the NIOSH-NCI investigators also highlight their quantitative exposure assessment as a key study strength, it is important to again note that major concerns have been raised regarding the NIOSH-NCI extrapolation methodology (see earlier discussion). Both Attfield et al. (2012) and Silverman et al. (2012) acknowledge the imprecision in their exposure estimates, but conclude that it is likely a source of non-differential misclassification of exposure (i.e. bias of risk estimates towards the null) rather than a source of systematic bias in exposure-response coefficients. However, no support is provided for such a conclusion, and studies such as Rhomberg et al. (2011) have demonstrated how similar imprecision in exposure estimates can result in bias to exposure-response curves.

Between the two papers, a large number of statistical models were employed, totaling over 400 statistical comparisons. It can thus be expected that some statistically significant associations would be observed. Among the reported statistically significant associations are some that can be characterized as inconsistent and unexpected findings. Some of these inconsistent findings have been noted above, including a greater SMR and steeper exposure-response slopes for surface-only workers compared to the more heavily exposed ever-underground workers. Attfield et al. (2012) fail to address the SMR findings and hypothesize that the differences in exposure-response slopes can be explained by greater exposure of surface workers to atmospherically-formed secondary pollutants like nitro-PAHs; however, they offer no empirical evidence in support of this hypothesis, which ignores the time duration and air movement and dispersion accompanying any secondary pollutant formation. Other unusual findings involve the apparent “plateau-ing” of risk at higher levels of estimated REC exposure and attenuation of smoking effects by REC; the NIOSH-NCI study investigators note that such effects have been observed in other occupational epidemiologic studies, but the explanations for these findings are not well-understood and could indicate possible selection bias or exposure misclassification (Stayner et al., 2003). Finally, it bears mentioning that several findings from Silverman et al. (2012) suggest control for smoking confounding may have been incomplete, including (1) differences in smoking intensity based on first-person worker interviews versus NOK interviews (e.g. for current smokers, 1% versus 6% were found to smoke two or more packs per day based on a sample of direct participant interviews versus NOK interviews, respectively); and (2) differences in smoking lung cancer risks by worker location (e.g. lung cancer risks for specific levels of smoking intensity were about three times higher for surface-only workers than ever-underground workers). Overall, the DEMS epidemiologic analyses represent major contributions to the DE-lung cancer epidemiologic literature; however, prior to weighing their causal implications, greater scrutiny is needed to ensure the correct interpretations of the voluminous body of statistical data and modeling, and to understand the potential biases introduced by the imprecise exposure estimates, the lag-time choices, and likely incomplete adjustment for potential confounders.

Moreover, the NIOSH-NCI findings are not consistent with those of prior epidemiologic analyses of underground miners. Although this literature is limited and far from definitive, the bulk of prior epidemiologic results for various underground mining cohorts do not provide strong evidence of a causal relationship between DE exposure and lung cancer (Hesterberg et al., 2006; Gamble, 2010), despite historically elevated DE exposures. The recent Neumeyer-Gromen et al. (2009) updated analysis of a cohort of 5800 German potash miners is one of the better-conducted studies of the relationship between DE exposure and lung cancer in underground miners. This study is considered to represent a significant improvement over the prior Säverin et al. (1999) analysis for this cohort, due to longer follow-up, the use of more stable statistical models, and adjustment for smoking (Gamble, 2010). As summarized in Table 4, this study reported a significantly decreased lung cancer SMR of 0.73 (95% CI: 0.57-0.93), but elevated, although imprecise, RRs for internal comparisons where DE exposure was dichotomized - e.g. RR = 1.28 (95% CI: 0.61-2.71) and RR = 1.50 (95% CI: 0.66-3.43) for the entire cohort and for a sub-cohort “with particularly accurate [DE] exposure measurement,” respectively. Several analyses were conducted to look for exposure-response trends in both the full cohort and the sub-cohort with cumulative DE exposure represented as either percentiles (tertiles, quintiles) or a continuous variable; some positive trends were observed, but none achieved statistical significance. As noted in Table 4, this study had some notable limitations, including considerable missing information on smoking status and possible exposure misclassification stemming from the use of TC as a DE exposure surrogate rather than the more specific EC indicator.

Other miner's studies that have explicitly considered DE exposure include the Johnston et al. (1997) retrospective cohort study of British underground coal miners (reviewed in Gamble, 2010) and the Bergdahl et al. (2010) retrospective cohort study of Swedish iron ore miners (summarized in Table 4), both of which were inconclusive as to a link between DE and lung cancer. Although focused on coal workers, the 1997 IARC review of “Coal Dust” concluded that the epidemiologic data point to a lack of association between lung cancer and coal mining (IARC, 1997). Specifically, IARC classified coal dust as a Group 3 carcinogen- i.e. “cannot be classified as to its carcinogenicity to humans.” This finding has relevance to the DE-lung cancer question, given the ubiquitous presence of diesel engines in European underground coal mines since the 1930s (Hesterberg et al., 2006). Although there are some uncertainties regarding the nature of DE exposures in these studies, these findings for other mining populations are thus at apparent odds with DEMS findings.

Two other epidemiologic studies summarized in Table 4 bear some discussion, given their recent publication. Olsson et al. (2011a) conducted a pooled analysis of 11 European and Canadian case-control studies of lung cancer (totaling >13,000 cases and >16,000 controls). They reported an elevated odds ratio (OR) of 1.31 (95% CI: 1.19-1.43) for the highest quartile of cumulative DE exposure versus unexposed, as well as significant exposure-response relationships for both intensity of DE exposure and duration of exposure *(p* value < 0.01). Using a JEM-based exposure assessment methodology similar to previous Canadian case-control studies (e.g. Parent et al., 2007), Villeneuve et al. (2011) examined the relationship between both DE and GEE exposure and lung cancer risk for 1681 incident lung cancer cases and 2053 population controls from eight Canadian provinces. They observed slightly elevated, but statistically nonsignificant, associations among workers “ever” exposed to DE relative to unexposed workers (OR = 1.06, 95% CI: 0.89-1.25) and for the highest tertile of cumulative lifetime DE exposure versus unexposed (OR = 1.12, 95% CI: 0.89-1.40). Villeneuve et al. (2011) observed statistically significant exposure-response trends for estimates of DE cumulative lifetime exposure for all lung cancers as well as for both squamous and large cell subtypes. Villeneuve et al. (2011) also assessed the relationship between occupational GEE exposures and lung cancer risk, observing a positive, but statistically non-significant, exposure-response trend for estimates of GEE cumulative lifetime exposure (e.g. an OR of 1.11 (95% CI: 0.88-1.39) for the highest tertile of cumulative lifetime GEE exposure versus unexposed).

**Figure 7:**
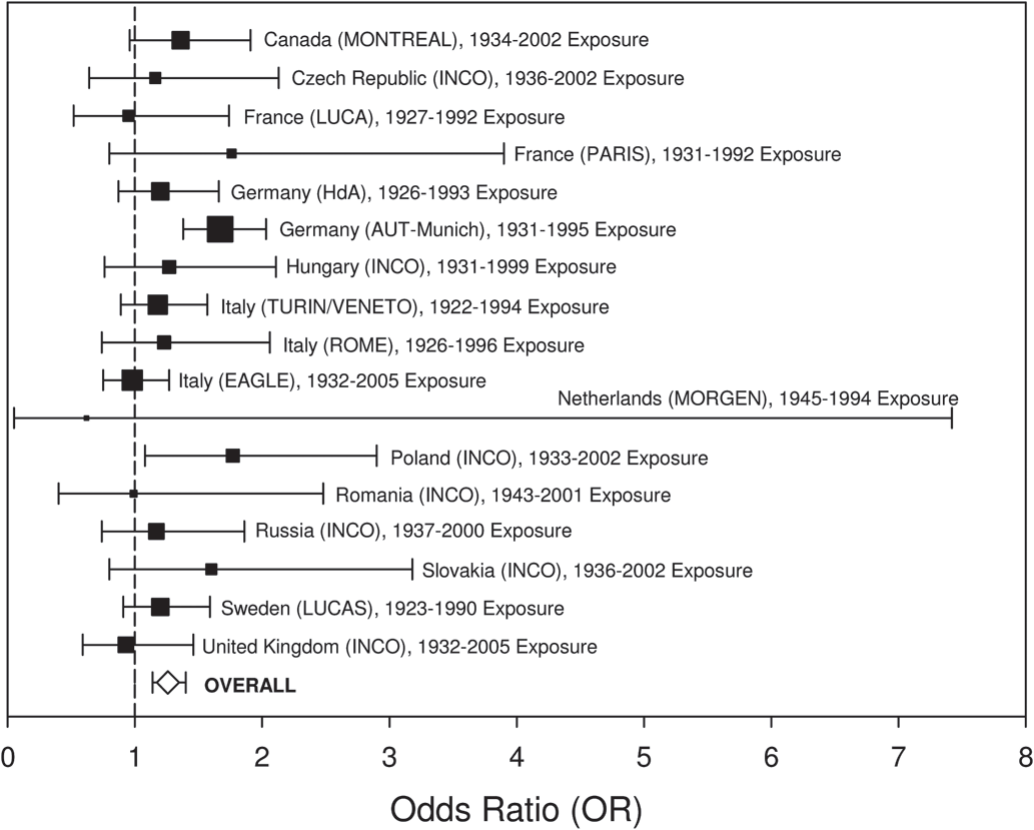
Chart shows study-specific and overall pooled-study lung-cancer odds ratios (OR) and 95% confidence intervals (CIs) for the highest quartile of cumulative diesel exhaust exposure compared with never-exposed, adjusted for age, sex, cigarette pack-years, time-since-quitting smoking, and ever-employment in a “List A” job (from Olsson et al., 2011a). Studies are identified by locations, with study acronyms provided in parentheses. As summarized in our Table 4, Olsson et al. (2011a) pooled information from 11 European and Canadian case-control studies covering 13,304 cases, with exposures typically between the 1920s/1930s and the 1990s/2000s. As noted in Olsson et al. (2011a), the symbol size reflects weighting from the random effects analysis. For global testing of the heterogeneity between the study ORs, Olsson et al. (2011a) reported an overall /-squared (I^2^) of 13.8% (p = 0.292) and concluded that there was no significant heterogeneity.

Compared to prior studies, both the Olsson et al. (2011a) and Villeneuve et al. (2011) studies have several notable strengths, including large sample sizes, control for smoking, and JEM-based semi-quantitative exposure assessments. Despite these various improvements to the study designs, the observed DE-lung cancer associations remained small and frequently lacked significance. Furthermore, it is important to consider some inconsistent findings and notable study limitations when weighing the findings from these studies (Table 4). In particular, Villeneuve et al. (2011) highlighted differences in the lung cancer excess risks between the two studies, hypothesizing that their findings of lower, and statistically non-significant, excess lung cancer risks may be due to more complete control for other potential occupational carcinogens such as silica and asbestos. As shown in Figure 7, Olsson et al. (2011a) observed substantial heterogeneity in lung cancer ORs for the individual datasets that they pooled together, including five study-specific ORs less than 1.0 and only two study-specific ORs that achieved statistical significance. Olsson et al. (2011a) concluded that the overall observed heterogeneity in the OR estimates was not significant (based on an *P* index of 13.8%, *p =* 0.292), although Morfeld and Erren (2012) raised concerns regarding the large influence of a single study (AUT-Munich) on the pooled study findings and the possible failure of a global test of heterogeneity to “provide reliable warning signals” when many individual study results are pooled into a large data set.

Several letters to the editor have expressed various concerns with the Olsson et al. (2011a) study related to study design, data reporting, and data interpretation (Bunn and Hesterberg, 2011; Morfeld and Erren, 2012; Mohner, 2012). Bunn and Hesterberg (2011) raised concerns regarding the possibility for exposure misclas-sification given the use of semi-quantitative, group-level assignments of exposure rather than actual DE exposure data, and for residual confounding due to incomplete corrections for smoking. In addition to their concerns regarding the large influence of the AUT-Munich study on the regression results, Morfeld and Erren (2012) also raised concerns regarding the possible effects of the exposure intensity scoring system used by Olsson et al. (2011a), whereby values of 0, 1, and 4 were used to represent no, low, and high DE exposures, respectively. They requested that a sensitivity analysis be conducted comparing results for other scoring systems such as a 0, 1, and 2-scheme. Finally, Möhner (2012) pointed out there was no adjustment for education despite the presentation of prior analyses by the Olsson et al. (2011a) study authors where there was adjustment for education and lower effect estimates were observed. Olsson et al. (2011b, 2012) provide the authors’ responses to these concerns; they discuss some additional data analyses, although some of the commenters’ requests for additional analyses were not addressed, such as regression results for models without the AUT-Munich study and a sensitivity analysis to explore possible effects of the 0, 1, and 4-scheme for scoring exposure intensity.

Overall, the evidence from a number of the recent epidemiologic studies is similar to that provided by prior studies, including both findings of small increased lung cancer risks and, in some cases, a lack of DE-lung cancer association (see Table 4). While the recently published NIOSH-NCI epidemiologic studies of miners (Attfield et al., 2012; Silverman et al., 2012) provide some strong evidence of exposure-response trends for both estimates of cumulative and average intensity REC exposure, inconsistencies in exposure-response relationships were observed between surface-only workers and ever-underground workers and between the two studies. In particular, Attfield et al. (2012) observed stronger exposure-response trends for surface-only workers versus ever-underground workers in the cohort study analyses, while in the nested case-control study analyses, Silverman et al. (2012) reported a general absence of increased lung cancer risk among surface-only workers, irrespective of the level of exposure. Moreover, both studies reported evidence of either a plateauing of the exposure-response relationship or a decrease in risk at high exposures. Although similar plateauing has been observed for high exposures in some occupational cohort studies, the specific explanations for these trends are not well-understood (Stayner et al., 2003). In addition, we have previously discussed the concerns that have been raised regarding the NIOSH-NCI exposure assessment methodology and the potential for large exposure mis-classification bias. While some additional studies (e.g. Garshick et al., 2008; Villeneuve et al., 2011; Olsson et al., 2011a) provide stronger evidence of an exposure-response relationship between various surrogates of DE cumulative exposure and lung cancer risk than previously available from older studies, some limitations and inconsistencies in findings from these studies have been noted. In addition, other well-conducted studies did not observe positive, statistically significant exposure-response trends (see Table 4 - e.g. Soll-Iohanning et al., 2003; Guo et al., 2004; Richiardi et al., 2006; Neumeyer-Gromen et al., 2009).

Adding to the uncertainty regarding a DE-lung cancer exposure-response relationship, consistent exposure-response trends are not apparent within occupational cohorts, such as railroad workers, trucking industry workers, and miners. This is a result of findings of either no excess risks, or small excess risks, for some job categories considered to have among the highest DE exposures (e.g. railroad shopworkers, truck mechanics, underground miners). In particular, Attfield et al. (2012) reported a lower lung cancer SMR for ever-underground workers than surface-only workers (1.21 versus 1.33), despite their data indicating that mean REC exposure levels were 31- to 167-fold higher for the ever-underground workers. Valberg and Watson (2000) previously demonstrated the absence of an apparent exposure-response trend for occupations with widely differing DE exposures (e.g. underground miners versus railroad workers versus truckers). The DEMS findings are consistent with the Valberg and Watson (2000) findings, as Attfield et al. (2012) reported an overall excess of lung cancer mortality (SMR = 1.26, 95% CI: 1.09-1.44) that is only marginally higher than what has been reported for other, much less-exposed worker populations (e.g. Laden et al. (2007) reported SMRs ranging from 1.08 to 1.16 for different categories of drivers in their study of unionized US trucking industry workers).

### Laboratory animal studies of TDE

We previously published a comprehensive review of the chronic inhalation carcinogenicity bioassays of DE from older-technology diesel engines (i.e. TDE) (Hesterberg et al., 2005). In addition, other in-depth reviews of these studies are also available that provide detailed summaries of the various DE chronic bioassays (Mauderly and Garshick, 2009; US EPA, 2002; IARC, 1989). As shown in Table 3, findings from the first series of large-scale (50 or more animals per group) lifespan bioassays of rats and mice were published in the mid- to late- 1980s. Additional rodent lifespan bioassays included inhaled DEP and carbon black particles, which are a form of EC that is nearly free of organics (Heinrich et al., 1995; Nikula et al., 1995). Since the mid-1990s, only a single large-scale, lifespan bioassay of inhaled DE has been conducted, namely the Stinn et al. (2005) nose-only inhalation study of male and female Wistar rats. All of the available large-scale lifespan bioassays in rats and mice were conducted using pre-1995 diesel engines, and generally 1980s-era light-duty engines, and thus relate to potential tumorigenic effects of TDE and not NTDE.

At the time of the 1988 IARC assessment, chronic bioassays had already been conducted in several animal species (rats, mice, hamsters, monkeys), with only lifetime exposure in rats providing consistent evidence of tumorigenic effects at highly elevated DE levels. Although it was hypothesized in the mid-1980s that the tumorigenic effects in rats may be the consequence of a “lung overload with particles” response rather than a direct genotoxic response of DEP mutagens (Vostal, 1986; Wolff et al., 1987), there was little understanding at this time of the overload mechanism in rats exposed to DE and its potential relevance to humans. In the last two decades, and primarily in the 1990s, there has emerged a paradigm shift in the scientific thinking regarding DE carcinogenic potential, away from a focus on DE chemical mutagens to a focus on the role of the particle and the species-specific response in rats to lung overload (HEI, 1995).

The lung overload phenomenon in rats exposed to protracted, highly elevated levels of DEP and other poorly-soluble nonfibrous particles (e.g. carbon black, titanium dioxide, talc, coal dust) has now been reviewed extensively in numerous publications (e.g. Oberdörster, 1995; Mauderly, 1996, 1997, 2000; Mauderly and McCunney, 1996; Valberg and Crouch, 1999; ILSI, 2000; US EPA, 2002; Hesterberg et al., 2005; Mauderly and Garshick, 2009). We previously summarized the current understanding regarding the apparent mechanism whereby lifetime inhalation of very high levels of DE leads to lung tumors in rats, namely, deposition of high levels of particles in the lungs results in an impairment of alveolar-macrophage (AM)-mediated lung clearance and, for deposition rates well in excess of clearance rates, the accumulation of excessive lung burdens of particles; excessive particle accumulation initiates an inflammatory response to which rats are particularly vulnerable; chronic inflammation, with ongoing release of oxygen free radicals from pulmonary macrophages and neutro-phils, damages lung tissues and stimulates tissue repair, increasing the chances of DNA transcription errors and failure of DNA repair mechanisms; at the same time, oxygen free radicals are released that can act as direct mutagens (Hesterberg et al., 2006).

**Figure 8:**
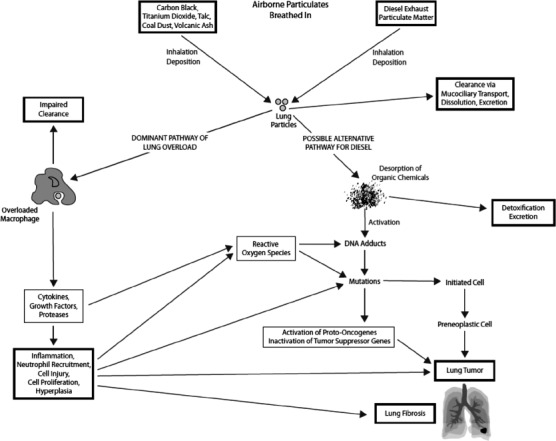
Possible mechanistic pathways leading to lung tumors in rats exposed by inhalation to protracted, high concentrations of poorly-soluble particles (adapted from Hesterberg et al., 2005 and HEI, 1995).

Figure 8 describes lung overload and its consequences in rats, and it updates prior figures in Hesterberg et al. (2005) and HEI (1995) that showed pathways by which inhaled, insoluble particles might lead to lung tumors in this species. Due to the evidence demonstrating similar rat lung tumor responses for a suite of poorly-soluble nonfibrous particles (e.g. DEP, carbon black, titanium dioxide, talc, coal dust), we have indicated lung overload to be the dominant mechanistic pathway in Figure 8. We have also included the pathway proposed in the late 1970s and early 1980s whereby organic compounds desorbed from DEP might induce lung tumors in rats from their direct genotoxic activity, although the body of evidence supports lung overload as the operative pathway in rats (Mauderly and McCunney, 1996). As reflected in Figure 8, the DE tumorigenic response in rats is now recognized to be a rat-specific, lung-specific process that is initiated only with protracted exposure to high levels of relatively insoluble particles and that is due to effects of particle-loading and not chemicals *perse.* As emphasized in Oberdörster (1995), impaired particle clearance is a key feature of the lung overload concept, as lung tumors and/or fibrosis have only been observed in rats for cases where lung burdens were sufficient to cause impaired particle clearance. Figure 9 from Wolff et al. (1987) shows experimental evidence of impaired lung clearance in rats resulting from both “high” (7.0mg/m^3^) and “medium” (3.5mg/m^3^) long-term DE exposures.

**Figure 9:**
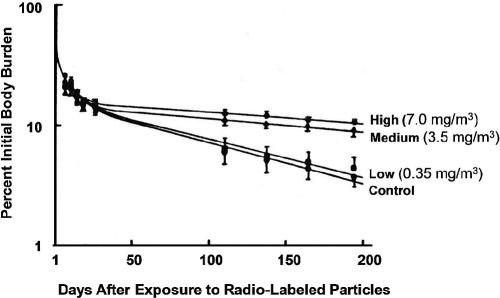
Impaired lung clearance in rats of ^134^Cs-radiolabeled particles inhaled after the end of 24-months DE exposure (for high, medium, and low DE exposure concentrations of 7.0,3.5, and 0.35 *\i%l* m^3^, respectively) and for a control population (Omg/m^3^ DE exposure). Data points are means ± standard errors (SEs). From Wolff et al. (1987).

Several key pieces of evidence that contributed to today's understanding of the lung overload concept in rats include: (1) findings from the body of DE chronic inhalation bioassays that demonstrated a significant excess of lung tumors only in rats exposed at high DEP levels and not in other species such as mice, hamsters, and guinea pigs (Mauderly et al., 1996; US EPA, 2002), (2) a lack of increased lung tumors in rats exposed to lower DEP levels (see more detailed discussion below regarding the evidence for an apparent threshold level for rat tumorigenicity), and (3) mid-1990s reports (Nikula et al., 1995; Heinrich et al., 1995) that similar lung overload responses occurred in rats exposed to elevated levels of mutagen-free carbon black. Moreover, Driscoll et al. (1996) reported findings supporting the role of an inflammatory pathway for lung tumor formation in rats following lifetime exposure to high levels of carbon black, showing that, under overload exposure conditions, the inflammation products increased mutation levels in alveolar epithelial cells.

**Figure 10:**
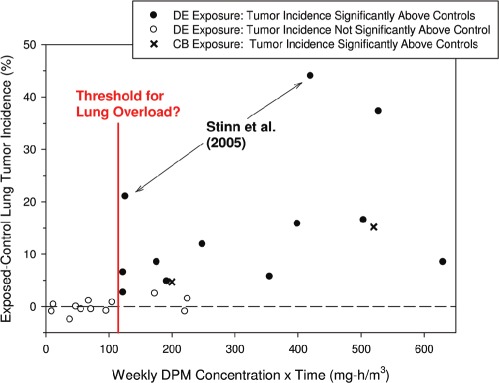
Relationship of normalized weekly exposure of rats to DEP versus rat lung tumor response (adapted from Mauderly and Garshick, 2009). Data from nine published studies with groups of 50 or more rats exposed >24 months to DE; data from the single chronic rat study published since the 1988 IARC DE review - Stinn et al. (2005) - are specifically labeled. Lung tumor increases are shown (exposed minus controls). Dashed line represents control incidence (no net increase). Open circles represent exposed groups with no statistically significant increase above the control incidence. Closed circles represent exposed groups with a statistically significant increase above individual control group lung tumor incidence. In addition to the DEP study data, we have also plotted data for carbon black (CB) from Nikula et al. (1995). Although Heinrich et al. (1995) also included a CB exposure group and observed a 27% excess in lung tumor incidence (exposed minus controls), we did not include this data point in the figure since the weekly exposure rate of 990 mg-h/m^3^ is well outside the range of DEP exposure rates and would have thus distorted the figure scale. (See colour version of this figure online atwww.informahealthcare. com/iht)

Figure 10 summarizes the consistently positive lung tumor responses that have been observed in rats for chronic, highly elevated DEP exposure conditions, showing the results normalized to a per week exposure rate (mg-h/m^3^). Based on similar analyses in Mauderly and Garshick (2009) and Hesterberg et al. (2005), this figure includes data from the nine large-scale, lifespan DE inhalation bioassays that have been conducted using rats. As discussed earlier, most of these bioassays have reported statistically significant increases in lung tumor incidence in rats at highly elevated DEP exposure levels, with the only exceptions being the Ishihara (1988) bioassay of a light-duty diesel engine and the Lewis et al. (1989) mine engine study. As shown in Figure 10, statistically significant increases in lung tumor incidence have only been observed in large-scale chronic rat bioassays when the weekly exposure rate has exceeded approximately 100 mg-h/m^3^, providing evidence of an apparent threshold exposure level for inducing lung tumors in rats. This figure further illustrates that statistically significant excesses in lung tumor incidence have also been observed for carbon black for weekly exposure rates exceeding 100 mg-h/ m^3^. As noted previously, carbon black is a poorly-soluble fine particle consisting of nearly pure EC with little organic content, including mutagens such as PAHs that are found in DEP from older diesel engines (Watson and Valberg, 2001).

Valberg and Crouch (1999) performed a meta-analysis of these data (all except those from the most recent Stinn et al. study), concluding that the data suggest a response threshold in the range of 200-600 μg DEP/m^3^ (note that a weekly exposure rate of 100 mg-h/m^3^ corresponds to an average continuous exposure level of about 600 μg/m^3^ -i.e. 106,000 μg/m^3^/week divided by 168 h/week). In Figure 10, the data from the recent Stinn et al. (2005) ratbioassay appear to stand out from other data, in particular those for the lower DEP dose (3 mg/m^3^; 126 mg-h/m^3^) which are close to the apparent tumor threshold level. Stinn et al. (2005) highlighted several differences between their study design and other chronic bioassays that may have contributed to an increased sensitivity, includingthe use of a high-longevity rat strain, restrained nose-only exposure conditions, and complete lung sectioning. Interestingly, Stinn et al. (2005) reported several observations that provide further support for the lung overload mechanism of tumorigenicity, including no significant increase in DNA adduct levels and evidence of both enhanced particle retention and progressive inflammation in the rat lungs.

The rat tumorigenic response is now generally agreed to be a species-specific threshold response of limited relevance to DE human carcinogenic risk, and consequently a consensus opinion has emerged that the rat data for lung overload conditions should not be used for estimating human lung cancer risks from DE inhalation (Mauderly 1997, 2000; Cal EPA, 1998; US EPA, 2002; Hesterberg et al., 2005). As concluded by US EPA in the Diesel HAD (US EPA, 2002), “Overload conditions are not expected to occur in humans as a result of environmental or most occupational exposures to DE. Thus, the rat lung tumor response is not considered relevant to an evaluation of the potential for a human environmental exposure-related hazard.” Prior to this, the Presidential/ Congressional Commission on Risk Assessment and Risk Management (Omenn, 1997) noted, “some chemicals elicit tumors in rodents only through mechanisms or at doses that have been clearly demonstrated to be very different from mechanisms and exposures in humans.” For such chemicals, the commission recommended against regulation as a carcinogen and extensive risk assessment.

There is no human evidence of a lung tumor response to particle overload conditions as observed for the rat, with coal miners serving as an illustrative example of a worker population subject to lung overload with both coal dust and possibly DEP (Oberdörster, 1995; ILSI, 2000; Hesterberg et al., 2005, 2006). This idea is illustrated by Table 5, which has been adapted from Oberdörster (1995). As discussed by Oberdörster (1995), there is indirect evidence of impaired lung clearance for coal workers from several studies (Freedman and Robinson, 1988; Freedman et al., 1988; Stober et al., 1965), and direct evidence of non-cancer pulmonary effects that are associated with high particle loads, including chronic pulmonary inflammation, pulmonary fibrosis, and localized emphysema. However, there is no evidence of a significantly increased risk of lung cancer among coal workers; as indicated previously, coal dust has been classified by IARC as a Group 3 carcinogen - i.e. not classifiable as to its carcinogenicity to humans (IARC, 1997). Moreover, Table 5 indicates that the rat is also a poor predictor of carcinogenicity in even similar species such as mice and hamsters under particle overload conditions. While impaired particle clearance has been observed in species other than rats (e.g. mice and hamsters), rats are the only species in which increased lung tumor formation has been observed.

**Table 5:** Summary of pulmonary effects in different species related to high particle load (from Oberdörster, 1995).

Pulmonary effect	Rat	Mouse	Hamster	Evidence in coal workers^a^
Prolonged particle clearance	++	++	++	[X]
Inflammation	++	+	(+)	X
Cell proliferation	++	+	(+)	X
Fibrotic foci	++	+/-	(+)	X
Localized emphysema	+	-	(-)	X
Tumors	++	-	-	-

Notes: Overall response to highly-insoluble, low-toxicity particles: rats>mice>hamsters; rats>primates (?).

The use of () for the hamster indicate that response is present but weaker than that observed for the rat and/or mouse.

a The evidence in coal workers (X) is not meant as a quantitative comparison to the three rodent species but merely indicates that a given adverse response has been observed in these workers. As detailed in the text, indirect evidence ([X]) in coal workers for prolonged lung clearance comes from studies by Freedman and Robinson (1988), Freedman et al. (1988), and Stober et al. (1965).

Overall, much has been learned about the overload mechanism in rats in the last two decades, both from the study of DE as well as through investigations of the rat response following inhalation of other low-solubility particle such as carbon black (Valberg et al., 2006). It is now widely accepted that the tumorigenic response observed in the chronic rat bioassays reflects a species-specific response to inhaled-particle overload conditions, rather than the direct genotoxic effects of DE mutagenic compounds. Importantly, the post-1988 animal study findings have consistently buttressed the lung overload concept for tumorigenic effects in the rat model that was in its infancy at the time of the 1988 IARC DE review, with no new studies offering counter explanations for the occurrence of lung tumors in rats chronically exposed to high TDE concentrations. There have been fewer animal studies of DE carcinogenicity in recent years, but findings from the small number of recent studies continue to provide support for the particular susceptibility of the rat lung to DE-induced tumors *via* the lung overload mechanism. For example, the previously mentioned Stinn et al. (2005) chronic rat bioassay reported evidence of both particle deposition and progressive inflammation in rat lungs, as well as no significant increase in DNA adduct levels. In addition, for a 6-month bioassay of strain A/J mice using four dilutions of whole emissions (DEP concentrations of 30, 100, 300, and 1,000 μg/m^3^) from a 2000-model-year HDDE, Reed et al. (2004) reported no statistically significant increases in either lung tumor incidence or multiplicity, or any evidence of an exposure-related trend. Although not a lifetime bioassay, Reed et al. (2004) used a lung tumor-prone strain of mice, and failed to detect any significant changes in two indicators of carcinogenic potential, namely proliferation of lung adenomas as well as micronucleated reticulocyte counts in peripheral blood.

### *In vitro* genotoxicity studies of TDE

As indicated in Table 3, the potential carcinogenicity of DE was first predicted in the late 1970s, early 1980s based on short-term bacterial mutagenicity assays of organic solvent extracts of DEP (Huisingh et al., 1978; Clark et al., 1981, 1984; Claxton, 1983; Schuetzle, 1983; Schuetzle et al., 1985; Schuetzle and Lewtas, 1986). Since this time, and particularly in the 1980s and early 1990s, the genotoxicity of both DEP extracts and whole DEP samples has been extensively evaluated in a number of *in vitro* bioassays using Salmonella bacteria and mammalian cell lines. Detailed reviews of these data are available (Claxton, 1983; Lewtas, 1983; Vostal, 1983; Lewtas and Williams, 1986; McClellan, 1987; IARC, 1989; Rosenkranz 1993, 1996; HEI, 1995, 1999; IPCS, 1996; Cal EPA, 1998).

In brief, there is a body of evidence supporting the *in vitro* genotoxicity of organic compounds extracted from DEP using strong organic solvents like dichloromethane, as well as some studies suggesting that DEP coated with surfactant may be genotoxic. There is a lesser amount of evidence supporting the genotoxicity of whole DE (Hesterberg et al., 2006). Various studies have demonstrated the mutagenicity of organic solvent extracts of DEP in several strains of *Salmonella typhimurium* with and without rat liver S9 activation (Huisingh et al., 1978; Claxton, 1983; Brooks et al., 1984) and Escherichia coli (Lewtas, 1983). Factors such as engine operating conditions and fuel type have been shown to influence the mutagenicity of DEP (McMillian et al., 2002; Kado et al., 2005). Studies have demonstrated the mutagenicity of DEP extracts in several mammalian cell lines including mouse lymphoma (Mitchell et al., 1981), Chinese hamster ovary (CHO) cells (Brooks et al., 1984; Morimoto et al., 1986), and human lymphoblast (Liber et al., 1981). Extracts of DEP were also observed to increase sister chromatid exchanges (SCE) in CHO cells (Mitchell et al., 1981; Brooks et al., 1984). DEP dispersed in an aqueous mixture containing dipalmitoyl lecithin, a component of pulmonary surfactant, produced increased responses in mammalian cell lines for SCE (Keane et al., 1991), micronucleus tests (Gu et al., 1992), and unscheduled DNA synthesis (Gu et al., 1994). Don Porto Carero et al. (2001) observed significant DNA damage in two human cell lines in the comet assay for both DEP extracts and washed DEP particles. Pereira et al. (1981) reported that inhalation exposure to DE for 7 weeks in mice produced increased incidences of micronuclei 6 months after exposure, while Sato et al. (2000) observed increased mutant frequency and DNA adducts in lung DNA for 4-week DE inhalation exposures (6mg DEP/m^3^) among Big Blue transgenic F344 rats (Sato et al., 2000).

While there is this body of *in vitro* genotoxicity data for collected, extracted DEP, there are several well-understood limitations to using these data for assessing DEP carcinogenic potential. These limitations include the non-physiological nature of the *in vitro* test conditions, where there is (1) the absence of the normal lung-defense mechanisms (e.g. macrophage mediated and mucociliary clearance), (2) the absence of cellular protective mechanisms, such as antioxidants and DNA repair, that act to prevent the expression of intracel-lular damage or DNA mutations, (3) the common use of hot organic solvents to obtain DEP extracts that can enhance the bioavailability of the organic compounds in DE compared to real-life *in vivo* conditions, and (4) the use of extremely high doses compared to what is deposited in the alveolar regions of the lung after inhalation. There remains some uncertainty regarding the fraction of DEP mutagens that is bioavailable in the lungs under environmental exposure conditions, but an increasing amount of data indicate that they are only poorly bioavailable in aqueous-based lung fluids (King et al., 1981; Leung et al., 1988; Bevan and Ruggio, 1991; HEI, 1995; Gerde et al., 2001; Borm et al., 2005; Hesterberg et al., 2005). The lesser evidence of elevated *in vitro* mutagenic activities observed for whole DEP samples (in contrast to solvent extracts of DEP) provides additional support for the poor *in vivo* bioavailability of DEP mutagens (HEI, 1995; Randerath et al., 1995). Lastly, recent studies have demonstrated formation of reactive artifacts - e.g. nitrated organic compounds - on filters during the collection of DEP mass for *in vitro* testing, suggesting some of the mutagenic activity observed in *in vitro* bioassays may be artifactual, i.e. due to chemicals created as a result of the sampling itself (Arey et al.,1988; Khalek, 2004; Hesterberg et al., 2005, 2006; Maricq, 2007).

Overall, while *in vitro* genotoxicity is widely regarded as an indicator of the mutagenic potential of a substance, it is recognized that mutagenicity correlates poorly with carcinogenic potential (Kamber et al., 2009). Specifically, Kamber et al. (2009) provide data showing the sensitivity of the Ames assay for predicting carcinogenicity to range from 58 to 63%, and the specificity to range from 50% to 63%. Moreover, as discussed previously, there is a lack of evidence for the direct genotoxic effects of DEP under the conditions of an inhalation bioassay. While it has been suggested that adduct formation following particle inhalation may be a non-specific PM response rather than a direct genotoxic response (Hesterberg et al., 2006), there is conflicting evidence regarding whether DE/DEP inhalation is associated with significant changes in levels of lung-cell DNA adducts in laboratory animals (Bond et al., 1990a, 1990b, 1990c; Randerath et al., 1995; Gallagher et al, 1994; Stinn et al., 2005). In addition, it is important to note that some recent studies provide evidence of similar *in vitro* mutagenic activities of extracts of contemporary GEE samples as for TDE extracts (Liu et al., 2005; Seagrave et al., 2002), but as discussed more later, the limited number of chronic inhalation bioassays of GEE have not generally observed any significant tumorigenic response of the lung (McDonald et al., 2007).

### Preliminary health effects data for NTDE

Currently, a limited number of laboratory animal and human clinical studies have investigated the potential health effects of DE that would meet the definition NTDE-i.e. DE from new and retrofitted advanced diesel engines utilizing multi-component emissions reduction systems (i.e. wall-flow DPFs, DOCs, and ULSD fuel) designed to meet the 2007 US EPA PM emission standard for on-road HDDEs (Hesterberg et al., 2011). As discussed earlier, there are no epidemiologic studies of NTDE exposures, nor is it anticipated that there will be epidemiologic findings specific to NTDE in the near future given that it will be some time before older diesel engine technologies are completely retired from use. None of the results available so far from NTDE health effects studies directly address the carcinogenic potential of NTDE. However, the need for research on the carcinogenic potential of NTDE was recognized prior to 2006, leading to the planning and design of the $20 million ACES emissions and toxico-logical testing of NTDE from diesel engines meeting the 2007/2010 emissions standards.

ACES, which is managed by HEI as a collaborative effort between industry and government, was designed to provide a wealth of emissions characterization and toxico-logical data for two groups of production-intent HDDEs, one meeting the 2007 US EPA PM and NO_x_ on-road HDDE standards and a second meeting the more stringent 2010 NO_x_ on-road HDDE standard (HEI, CRC, 2006). The ACES research program has three main components, including the Phase 1 emissions characterization of four 2007-model year engines (a Caterpillar C13, a Cummins ISX, a Detroit Diesel Corporation Series 60, and a Volvo Mack MP7), the Phase 2 emissions characterization of engines and control systems meeting the 2010 standards (i.e. those meeting the new stricter federal standards for NO_x_ emissions), and the Phase 3 animal exposure studies of NTDE from 2007-compliant engines. The ACES working hypothesis is that “Emissions from combined new heavy-duty diesel engine after-treatment, lubrication and fuel technologies designed to meet the 2007 NO_x_ and PM emission standards will have very low pollutant levels and will not cause an increase in tumor formation or substantial toxic health effects in rats and mice at the highest concentrations of exhaust that can be used (based on temperature and NO_2_ or CO levels) compared to animals exposed to ‘clean air,’ although some biologic effects may occur” (HEI, CRC, 2006). This hypothesis is based on the expectation that PM concentrations in NTDE will be well below concentrations producing lung tumors in rats via an overload mechanism. Thus, the rat bioassay of NTDE will serve to establish whether NTDE contains chemical species, including any formed inadvertently in the exhaust aftertreatment system, at sufficient concentration and potency to yield a carcinogenic response.

As reported in Khalek et al. (2011) and discussed earlier, the Phase 1 emissions testing has provided a comprehensive dataset that distinguishes NTDE from TDE. The Phase 2 emissions testing commenced in early 2012, and the Phase 3 animal exposure studies initiated in 2010 remain ongoing. Mouse and rat bio-screening studies are core components of the Phase 3 efforts, which also included the development and characterization of the exposure atmospheres at Lovelace Respiratory Research Institute (LRRI) (Mauderly and McDonald, 2012). These studies are expected to provide a suite of data relevant to evaluating the potential carcinogenic hazard and potential non-cancer health effects of NTDE, with evaluations of pulmonary function, necropsy, hematology, serum chemistry, bronchoalveolar lavage, lung epithelial cell proliferation, and histopathology (Mauderly, 2010). One- and three-month animal exposure studies, which included inhalation exposures to three dilutions of whole NTDE emissions (approximately 25:1, 115:1, and 840:1 that were set to achieve 4.2, 0.8, and 0.1 ppm NO_2_ concentrations) and a clean air control, have been completed and are described in HEI Report 166 (HEI, 2012); this three-part report includes detailed reports by McDonald et al. (2012), Bemis et al. (2012) and Hallberg et al. (2012).

The 30-month chronic rat bioassays, which were begun in 2010, include a long-term carcinogenesis bio-assay using Wistar Han rats; this bioassay was designed based on both the standard NTP bioassay, where a duration of two years is typical, and recognition that key bioassays of TDE (e.g. Mauderly et al., 1987) were conducted with 30-month exposures. The basis for the selection of the Wistar Han rat strain is documented in HEI Report 166 (HEI, 2012), along with the basis for selection of the exposure conditions. The Mauderly et al. (1987) study of TDE involved exposure of rats to diluted exhaust from a 1980 General Motors engine for 7 h/day, 5 days/week for 30 months. The lowest dilution of TDE exhaust in the Mauderly et al. (1987) study was 10:1, which yielded chamber atmospheres containing about 7000 μg/m^3^ of PM and a NO_2_ concentration of 0.7 ppm. Recognizing that the PM concentrations in NTDE from 2007-compliant engines would be quite low and that it was desirable to maximize exposure of the animals, HEI decided to conduct exposures with diluted exhaust for 16 h/day, 5 days/week for up to 30 months, if the survival of animals permitted. It was viewed desirable to maximize the PM exposures consistent with any limitations posed by other toxic agents in the exhaust, such as CO or NO_2_. The basis for selection of the dilution ratios and resulting exposure concentrations are discussed in HEI Report 166 (HEI, 2012) and McClellan et al. (2012).

The lowest dilution ratio in the ACES rat study (25:1), and thus the highest concentration of all exhaust constituents, was selected based on the Maximum Tolerated Dose (MTD) of NO_2_. This concentration was selected based on an earlier chronic NO_2_ exposure study conducted by Mauderly et al. (1989, 1990). In that study, rats were exposed to an NO_2_ atmosphere of 9.5 ppm for 7 h/day (66.5 ppm-h exposure) for 5 days/week for 24 months. This NO_2_ exposure produced the hallmark lesion of oxidant gas exposure - “mild hyperplasia of the epithelium in terminal bronchioles and an extension of bronchiolar epithelial types into proximal alveoli, giving the appearance of respiratory bronchioles.” In the ACES rat study, the 25:1 dilution ratio corresponds to a target NO_2_ concentration of 4.2 ppm. For a 16h/day exposure, this yields a 67.2 ppm-h exposure that is very closely matched to the 66.5 ppm-h exposures of Mauderly et al. (1989, 1990). As a MTD, it was thus expected that the 25:1 exhaust dilution used in the ACES rat study would produce pulmonary lesions similar to those observed with NO_2_ exposure (Mauderly et al., 1989, 1990). The two lower dilution ratios, 115:1 and 840:1, were selected to provide levels at which NO_2_-induced effects would probably not be observed.

Very recently, HEI released a three-part report (HEI, 2012) that includes detailed investigator reports (McDonald et al., 2012; Bemis et al., 2012; Hallberg et al., 2012) describing the subchronic exposure results for the ACES rat bioassays of NTDE from a 2007-compli-ant HDDE. The measured NO_2_ exposure concentrations were reported as 3.6±1.2, 0.95±0.57, and 0.11 ±0.12 ppm for the three diluted exhaust exposure groups, while PM concentrations (chamber inlet) of 13 ±5.7, 4 ±4, and 2 ± 6 μg/m^3^ were reported. For groups of male and female rats euthanized after 1, 3, and 12 months and groups of male and female mice euthanized after 1 and 3 months, McDonald et al. (2012) reported findings for over 100 biologic response variables addressing a diverse array of biological endpoints, including histopathologic (multiple tissues, including the airways), hematologic (several cell types, plus coagulation), serum chemistry (including triglyceride and protein components), lung lavage (including numbers of cells and levels of multiple cytokines and markers of oxidative stress), and pulmonary function (rats only). Overall, for the majority of biological response variables, no significant differences were observed between DE exposures and clean air controls. As was anticipated given the NO_2_ exposure concentrations at the MTD, mild histologic changes were observed in the respiratory tracts of rats (but not mice) after 3 months of exposure. Although there was evidence of progression of these histologic changes at 12 months (meaning that they were more widespread within the lung and in more animals), they were still scored as mild. Importantly, McDonald et al. (2012) concluded that these histologic changes were consistent with those observed in prior chronic bioassays of NO_2_ (e.g. Mauderly et al., 1989, 1990). In its commentary on the study, the HEI Review Committee expressed the same view (HEI, 2012).

Bemis et al. (2012) and Hallberg et al. (2012) conducted *in vivo* assessments of genotoxicity in both rats and mice from the 1-month and 3-month exposures to NTDE, investigating micronuclei formation in peripheral blood reticulocytes and markers of oxidative damage-related DNA damage and lipid peroxidation. Both teams of investigators concluded that no evidence of geno-toxic effects could be detected, although it is important to consider both the small group sizes used in these assessments (only five animals of each sex per exposure group) and that the assessments of genotoxicity only extended through 3 months of exposure. The HEI Review Committee Commentary concurred that the results obtained after 3 months of exposure to NTDE indicated an absence of genotoxicity (HEI, 2012).

The ACES rat exposures are continuing with 200 rats in each group being observed for up to 30 months of exposure. This long-term follow-up maximizes the potential for observation of any carcinogenic response related to NTDE exposure, the core objective of the ACES study.

**Figure 11:**
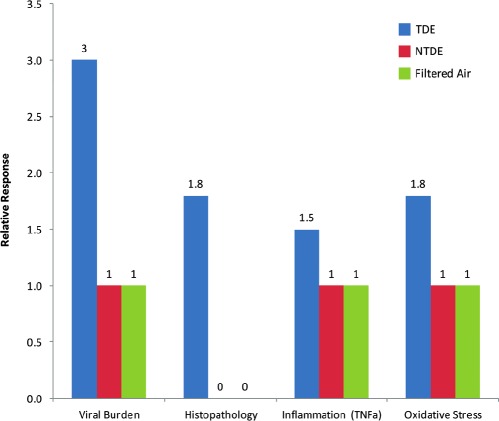
Summary of McDonald et al. (2004b) findings on the relative toxicity in mice of acute inhalation exposures (6h per day over 7 days) for a baseline uncontrolled, TDE emissions case (approximately 200 *\ig/m^3^* DEP) versus an emissions reduction case (low-sulfur fuel, catalyzed ceramic trap, 7 ng/m^3^). Expressed as relative responses to filtered air, findings are shown for four indicators of acute lung toxicity, namely respiratory syncytial virus (RSV) resistance, histopathology, lung inflammation (specifically, measurements of tumor necrosis factor-a (TNF-a)), and oxidative stress. (See colour version of this figure online at www. informahealthcare.com/iht)

Although limited, other laboratory animal and human clinical studies have investigated the potential acute effects of short-term NTDE exposures (e.g. McDonald et al., 2004b; Tzamkiozis et al., 2010; Lucking et al., 2011) and also provide preliminary evidence of the toxico-logical differences between NTDE and TDE. Figure 11 summarizes findings from the McDonald et al. (2004b) laboratory animal study that provides some of the more comprehensive health effects data available for NTDE. This study investigated a suite of sensitive measures of acute lung toxicity in mice, including lung inflammation, respiratory syncytial virus (RSV) resistance, and oxidative stress. As shown in Figure 11, the biological responses observed for a baseline TDE case were either nearly or completely eliminated for the NTDE case, where a catalyzed ceramic trap and low-sulfur fuel were used with the test engine (a Yanmar single-cylinder diesel engine generator). Hesterberg et al. (2011) provides an extensive review of the preliminary health effects data currently available for NTDE, showing the mounting evidence for the elimination of biological responses previously observed for TDE exposures.

It is also important to note that a limited amount of *in vitro* toxicity testing has also been conducted for NTDE, including mutagenicity testing. As reviewed in Hesterberg et al. (2011), a few studies have assessed the mutagenic potential of PM samples from bus exhausts considered to be NTDE versus TDE, as well as from compressed natural gas (CNG) buses (Kado et al., 2005; Kado and Kuzmicky, 2003; Nylund et al., 2004). Using bacterial mutagenicity tests (Salmonella/microsome tests), these studies provide evidence of highly-reduced mutagen emissions (i.e. numbers of revertant bacteria per vehicle distance traveled - e.g. krev/mile) for NTDE from DPF-equipped buses compared to both TDE and CNG exhaust. These studies have generally observed an increase in specific mutagenic activity (SMA, defined as the number of revertant bacteria per unit mass of PM collected - e.g. rev/μg PM) for NTDE compared to TDE, although generally lower SMA values for NTDE than for CNG exhaust. It is again important to emphasize that, given the well-recognized limitations of *in vitro* genotoxicity studies mentioned above, these *in vitro* mutagenicity results are of uncertain relevance to the carcinogenic risks posed by NTDE to humans. In particular, the California Air Resources Board (CARB, 2002) has noted the problems with the reliability of mutagenicity test results for cancer risk assessment: “The mutagenicity results are only an indication of the presence of potentially carcinogenic compounds in the samples analyzed. Although significant differences are an indication of relative toxicity potential of the samples analyzed, these results cannot be used to quantify cancer risk."

### NTDE versus GEE

**Figure 12:**
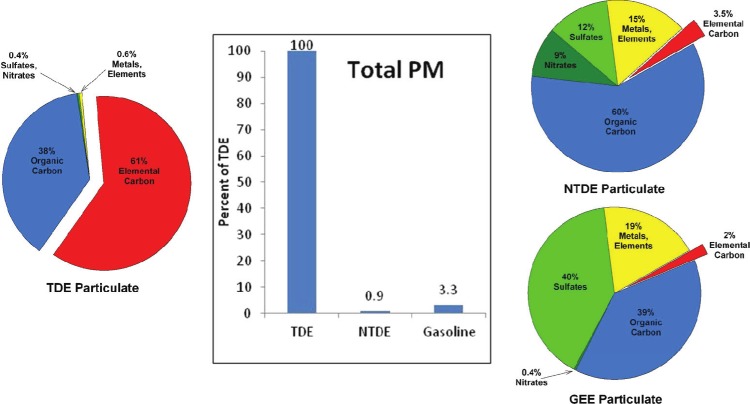
Comparison of total PM emissions (on a mass per-distance-traveled basis) and PM composition for light-duty automobile engine exhausts representative of TDE, NTDE, and GEE. All data based on particle composition measurements from Cheung et al. (2009), who conducted emissions testing on a chassis dynamometer for light-duty vehicles operated using different aftertreatment configurations and a cold-start New European Driving Cycle (NEDC) and a series of Artemis cycles. Specific vehicle configurations include a Euro 4+ Honda Accord (2.2 L, i-CDTi) equipped with a ceramic-catalyzed diesel particulate filter (c-DPF), a closed-coupled oxidation catalyst (pre-cat), and exhaust gas recirculation (EGR), operated using low sulfur (< 10 ppm) diesel fuel and lube oil with a sulfur content of 8900 ppm wt (considered to be NTDE); a Euro 3 Toyota Corolla (1.8 L) equipped with a three-way catalytic converter and operated using unleaded gasoline with a research octane number (RON) of 95 and fully synthetic lube oil (considered to be GEE); and a Euro 1 compliant Volkswagen Golf (TDI, 1.9 L) operated using diesel fuel with a nominal sulfur content of 50 ppm (considered to be TDE). (See colour version of this figure online at www. informahealthcare.com/iht)

As we have discussed previously (Hesterberg et al., 2011; McClellan et al., 2012), a convincing case can be made that the PM in NTDE shows a greater resemblance to particu-late emissions in contemporary GEE (i.e. GEE from modern gasoline engines equipped with three-way catalytic converters and operated using unleaded, low-sulfur gasoline) than TDE. As illustrated by Figure 12, such a determination can be based on the major changes in both PM mass emissions and composition in NTDE. Recognizing that emissions from specific engines/technologies can vary depending on a number of factors including engine specifications, fuel, operating cycle, sampling techniques, etc., Figure 12 shows emissions testing data from the recent Cheung et al. (2009) study of several combinations of light-duty vehicles and emissions control configurations. As shown in Figure 12, the lowest PM emissions observed in the Cheung et al. (2009) study were for the diesel vehicle with exhaust that can be classified as NTDE. In addition, the diesel vehicle with NTDE emissions was found to have a PM composition - consisting primarily of nitrates, sulfates, and OC species rather than the EC particles that dominate TDE- that more closely matched that of GEE from a Euro 3 - compliant car equipped with present-day aftertreatment technology typical of gasoline cars in the US and Europe (e.g. a three-way catalytic converter, leaded gasoline) than TDE.

Although there is certainly a need for chronic inhalation bioassay data that are specific to NTDE (i.e. the forthcoming ACES data), a case can be made as to the reasonableness of extrapolating the findings from chronic inhalation bioassays of contemporary GEE to draw preliminary conclusions regarding the possible carcinogenic potential of NTDE. There is a lack of chronic inhalation bioassay studies of contemporary GEE, but older studies of GEE, most conducted using 1970s and earlier engines and fuels, do not provide evidence of increased lung tumor formation (McDonald et al., 2007). These older GEE studies include the 1980s Battelle-Geneva study (Brightwell et al., 1986,^1989^) where groups of both rats and hamsters were exposed for 16 h per day, 5 days per week, for 2 years to the exhaust emissions from two Renault R18 1.6-liter gasoline engines, equipped with and without three-way catalytic converters and operated with unleaded gasoline. In addition, the Fraunhofer Institute conducted a chronic inhalation bioassay for GEE in the early 1980s where Wistar rats and Syrian golden hamsters were exposed for 19 h per day, 5 days per week, for 91 weeks to the exhaust from a 4-cylinder Volkswagen engine operated using leaded fuel (Heinrich et al., 1989; McDonald et al., 2007). Although detailed histopathological findings have not been published in the open literature for this study, Heinrich et al. (1989) noted that no significant increases in lung tumors were observed in rats for GEE exposures. In part due to the widespread recognition that contemporary GEE is cleaner than the GEE from 1970s and 1980s engines and fuels, additional chronic inhalation bioassays of GEE have not been conducted.

Although limited details on the exposure atmospheres are available in the open literature for both the Battelle-Geneva and Fraunhofer Institute GEE studies, the available data show some parallels between these studies and the ongoing ACES chronic inhalation bioassay. In particular, there are similarities in emissions dilutions (27:1 for the high-exposure group in both of the GEE studies versus 25:1 for the high-exposure group in the ACES NTDE study) and lower-level PM exposure concentrations (73 μg/m^3^ and <210 μg/m^3^ for the high-exposure groups in the Fraunhofer Institute and Battelle-Geneva studies, respectively, versus approximately 10 μg/m^3^ for the high-exposure group in the ACES NTDE study).

### Overview of prominent hazard assessments of TDE

Table 6 summarizes key conclusions from prominent hazard assessments conducted for DE and/or DEP by regulatory agencies and authoritative bodies. IARC reviewed DE in 1988, classifying DE as a Group 2A “probable” human carcinogen based on “limited” evidence for the carcinogenicity of DE in humans, but “sufficient” evidence in animals for both the carcinogenicity of whole DE and DEP extracts. IARC concluded that there was “inadequate” evidence for the carcinogenicity of gas-phase DE constituents in experimental animals based on studies showing a lack of increased tumor induction in rats and hamsters exposed to filtered DE. Although not shown in Table 6, IARC (1989) classified GEE as a Group 2B “possible” human carcinogen based on inadequate evidence for the carcinogenicity of whole GEE in humans and experimental animals, but sufficient evidence for the carcinogenicity of condensates/extracts of GEE in experimental animals. Among other hazard assessments, the World Health Organization's International Programme on Chemical Safety (WHO IPCS) classified DE in 1996 as “probably carcinogenic,” the US National Toxicology Program (NTP) classified DEP in 2000 as “reasonably anticipated to be a human carcinogen,” and US EPA classified DE in 2002 as “likely to be carcinogenic to humans.”

**Table 6:** Summary of DE/DEP hazard assessments conducted by regulatory agencies and authoritative bodies.

	Key Conclusions			
Regulatory Agency/Authoritative Body, Date	*Animal Evidence*	*Human Evidence*	*Overall DE/DEP Classification*	Quantitative Risk Assessment Performed?
NIOSH, 1988	“Confirmatory” for carcinogenesis	“Limited”	DE classified as “potential occupational carcinogen”	No
IARC, 1989	“Sufficient” for carcinogenicity of whole DE and DEP extracts; “inadequate” for gas-phase DE (with particles removed)	“Limited”	Whole DE classified as a “probable” human carcinogen (Group 2A)	No
IPCS, 1996	Rat data supportive of DE carcinogenicity	“Four most informative studies” supportive of increased risk for lung cancer; however, “no human data suitable for estimating unit risk”	DE “probably carcinogenic” to humans based on human epidemiologic evidence	Yes, using rat data; geometric mean inhalation cancer unit risk of 3.4 × 10^5^ per mg/m^3^ for bioassay-based estimations
California EPA, 1998	Rat data “have demonstrated” carcinogenicity of DEP	Supportive of causal association of DE and lung cancer as “reasonable and likely explanation”	Designated diesel particulate matter a “toxic air contaminant”	Yes, using human epidemiologic data; derived inhalation cancer unit risk of 3 × 10^−4^ per mg/m^3^ (expressed in terms of DEP)
NTP, 2000	Along with mechanistic data, viewed as supporting data	Provide evidence of elevated lung cancer risk in DE-exposed occupational groups	DEP classified as “reasonably anticipated to be a human carcinogen”	No
US EPA, 2002	Rat and mouse data for non-inhalation routes of exposure provide “supporting evidence of DPM's carcinogenicity and associated DPM organic compound extracts; chronic inhalation rat data viewed as not being predictive of a human hazard at lower environmental exposures	“Strongly supportive” of a DE-lung cancer causal relationship, but “less than that needed to definitively conclude that DE is carcinogenic to humans”	DE emissions classified as “likely to be carcinogenic to humans”	No
ACGIH, 2003			DEP Threshold Limit Value (TLV) and carcinogen classification withdrawn	No

As shown in Table 6, these groups have generally classified DE/DEP as a likely or probable carcinogen based on evaluations of the epidemiology and the experimental evidence from animal and *in vitro* studies, although most have concluded that the available health effects evidence is inadequate to support a quantitative risk assessment. While current in the sense of not havingbeen superseded by more recent assessments, the majority of these hazard assessments were conducted more than 10 years ago, well before the full-scale implementation of multi-component aftertreatment systems for on-road HDDEs and the emergence of NTDE. Their conclusions regarding DE carcinogenicity are thus based on pre-2000 health effects studies that focus on DE from pre-1988 diesel engines; in other words, they are specific to TDE, but not to NTDE. In a similar fashion, much of the health effects evidence relied upon by IARC in 1988 in its evaluation of GEE was also for older engines and fuels not representative of today's modern engines and fuels, including engines operating on leaded gasoline and lacking the modern three-way catalytic converters.

Given the emissions characterization data demonstrating the significant chemical, physical, and mass-emission differences between NTDE and TDE, it is clear that hazard assessments conducted using health effects studies of TDE are of questionable relevance to NTDE (Mauderly and Garshick, 2009; Olsson et al., 2011a; Hesterberg et al., 2011). Even prior to the full-scale emergence of NTDE, US EPA recognized in 2002 that their conclusions regarding DE health effects, as based on studies of older diesel engine technologies, may not apply to the DE from newer technology engines: “A notable uncertainty of this assessment is whether the health hazards identified from studies using emissions from older engines can be applied to present-day environmental emissions and related exposures, as some physical and chemical characteristics of the emissions from certain sources have changed over time. Available data are not sufficient to provide definitive answers to this question because changes in DE composition over time cannot be confidently quantified, and the relationship between the DE components and the mode(s) of action for DE toxicity is/are unclear.” There necessarily remain questions regarding the specific hazards posed by various DE components and the mode(s) of action for DE toxicity, in particular at lower levels of exposure typical of environmental and most occupational exposures; however, as discussed earlier, there is now an accumulated body of data characterizing the major differences in DE composition between NTDE and TDE.

Two major carcinogenic hazard assessments for DE are now pending, where it is expected that the extensive body of emissions characterization data and preliminary health effects findings for NTDE will be considered. IARC is scheduled to reevaluate DE, along with GEE and some nitroarenes, in June 2012. In addition, the US NTP announced in January 2012 that DEP was among 12 substances nominated for possible review in a future edition of the Report on Carcinogens (NTP, 2012).

### Concluding remarks on DE health effects research

The carcinogenic potential of DE from older diesel engine technologies (i.e. TDE) has been studied repeatedly using a wide variety of methodologies. However, despite the vast amount of health effects data generated to date, there remains controversy regarding whether the data are sufficient to support a causal, quantitative link between people inhaling occupational or environmental TDE and increased lung cancers. In addition, many uncertainties remain unresolved, including whether ambient DE exposure levels pose any excess lung cancer risk, whether specific chemical constituents in DE are key to carcino-genesis in humans, and what mechanisms could lead to DE-induced lung cancer at non-particle-overload conditions (Mauderly and Garshick, 2009; Ward et al., 2010).

Although there is now a sizable number of epidemiologic studies, recent studies continue to be affected by many of the same limitations and weaknesses as older studies, including a lack of actual DE exposure data, inadequate control of potential confounders, and findings of low-level risks (e.g. see Figure 7) that are difficult to interpret. The recently published NIOSH-NCI epidemiologic analyses of miners have some notable strengths compared to prior DE-lung cancer epidemiologic studies (e.g. a large cohort size, adequate latency, high levels of DE exposure, etc.), but as discussed previously, also have limitations and uncertainties. There will no doubt continue to be disagreements regarding whether the evidence is sufficient to support a causal, quantitative link between DE exposure and lung cancer risk. Moreover, it should not be forgotten that even the most recent DE epidemiologic studies apply only to historical exposures to TDE, and not to present and future exposures that involve NTDE.

At present, only a very limited amount of data are available to compare the biological responses from NTDE from new and retrofitted advanced diesel engines to those from TDE. However, these preliminary data support the idea that NTDE is toxicologically distinct from the TDE from older engines, with the particulate emissions in NTDE likely more similar to those in contemporary GEE and CNG exhaust than TDE (Hesterberg et al., 2011). There are currently neither epidemiologic data nor *in vivo* toxicology data directly bearing on NTDE carcinogenic potential, although it has been hypothesized that the major reductions in the mass emissions and changes in chemical composition of PM in NTDE will contribute to diminished NTDE carcinogenic potential compared to TDE (HEI, CRC, 2006). The ACES chronic bioassays are expected to contribute important findings regarding the carcinogenic potential of NTDE.

## Discussion and conclusions: recommendations on the path forward

This review has demonstrated the historical interplay between the DE emissions characterization and exposure assessment efforts, the DE health effects research, and the evolution of diesel emissions regulations. Together with technological innovation, each contributed to the emergence of NTDE. As summarized in this paper and discussed in greater detail in Hesterberg et al. (2011), there is now a sufficient body of data, not only from emissions characterization studies, but also from a limited number of health effects studies, that distinguish NTDE from TDE. Compared to TDE, PM levels have been reduced approximately 100-fold in NTDE, and similarly large reductions have also been achieved for numerous other DE particulate and gaseous species, including mutagens such as PAHs and nitro-PAHs. The limited health effects studies of NTDE provide evidence that some of the adverse health effects observed for TDE are not observed with NTDE (e.g. adverse vascular and prothrombotic effects, based on findings from Lucking et al., 2011; several measures of acute lung toxicity commonly used in short-term rodent bioassays, including lung inflammation, RSV resistance, and oxidative stress, based on McDonald et al., 2004b).

In short, there is now a critical mass of data differentiating NTDE from TDE and supporting the idea that future DE hazard assessments should evaluate NTDE and TDE separately (McClellan et al., 2012). This idea of distinctly separate hazard assessments for NTDE and TDE is not a new concept, having been proposed by US EPA back in 2002 in the Diesel HAD, based on data indicating different characteristics (e.g. reduced amounts of adsorbed organics on carbon particles) between pre-1990 diesel engines that were the predominant focus of the available DE health effects studies and then-contemporary diesel engines (US EPA, 2002). Due to the continued innovation in diesel engine technologies and the full-scale implementation of multi-component aftertreatment systems among on-road HDDE, there are even greater differences between the emissions from present-day on-road HDDEs and pre-1990 diesel engines, along with a sizable body of data characterizing the quantitative and qualitative differences between NTDE and TDE (Hesterberg et al., 2011). Furthermore, both mass emissions and chemical composition data show that the PM in NTDE has a greater resemblance to the PM in contemporary GEE than in TDE (Cheung et al., 2009; Hesterberg et al., 2011). Hesterberg et al. (2008, 2011) previously demonstrated greater similarities in PM emissions from post-2006 on-road HDDEs to the PM emissions from CNG buses than to those in TDE. By inference, combining NTDE with TDE in a DE hazard assessment can be viewed as analogous to combining GEE or CNG exhaust with TDE. IARC and other agencies have traditionally conducted separate hazard assessments for engine exhausts from different types of internal-combustion technologies, including DE and GEE.

Given its plans to reevaluate DE (along with GEE and some nitroarenes) in June 2012, IARC will be the first authoritative body to assess DE carcinogenic health hazards since the emergence of NTDE. As discussed in this paper, much has changed since the last IARC review of DE in 1988. For its upcoming reevaluation of DE, IARC has available not only the sizable body of data distinguishing NTDE from TDE, but also two more decades of study on the carcinogenic potential of TDE, as discussed in this review. Prior to commenting on the potential implications of these additional data to the upcoming IARC review of DE, it may be helpful to first briefly describe the IARC classification system and how it has changed since 1988.

In providing qualitative scientific judgments on the evidence for or against the carcinogenicity of environmental factors, IARC weighs the body of health effects evidence from epidemiologic studies, animal bioassays, and mechanistic studies. As described in greater detail in the IARC Preamble (IARC, 2006) and various reviews (e.g. Cogliano et al., 2008), the IARC classification system uses four carefully defined category descriptors to assess the strength of evidence from human and animal studies: “*sufficient* evidence of carcinogenicity',’ “*limited* evidence of carcinogenicity',’ “*inadequate* evidence of carcinogenicity',’ and “evidence suggesting lack of carcinogenicity.” When reviewing the scientific evidence so as to determine the appropriate category descriptor, IARC considers study quality, for example, in epidemiological studies, the possible roles of bias, confounding, and chance. For epidemiological studies, “sufficient” evidence of causality generally requires: (1) a strong association (e.g. a large relative risk) that is replicated in several studies with similar designs; (2) risks that increase with exposure; (3) observed temporality; (4) precision; (5) biological plausibility; and (6) reasonable confidence that chance, bias, and confounding have been ruled out. In assessing the strength of evidence from animal studies, IARC considers: experimental conditions (e.g. route and duration of exposure, species, sex, age, follow-up, etc.), the consistency of the results (e.g. across species or target organs), the spectrum of the neoplastic response (e.g. benign versus malignant tumors), and the possible role of modifying factors. The strength of mechanistic information is also assessed, in particular relating to whether a mechanism yielding tumors in animals is also relevant to humans. Ultimately, IARC's evaluations of the human, animal, and mechanistic evidence are combined into a classification of an agent being either: carcinogenic to humans (Group 1), probably carcinogenic to humans (Group 2A), possibly carcinogenic to humans (Group 2B), not classifiable as to its carcinogenicity to humans (Group 3), and probably not carcinogenic to humans (Group 4).

Perhaps the most significant change to the IARC process since 1988 involves IARC's efforts to better integrate mechanistic evidence into its classification process. Specifically, in 1991, IARC assembled a working group to provide advice on how mechanistic information should be used to inform the overall evaluation of carcinogenicity to humans, addressing in particular the question of extrapolation of animal study findings to predicting cancer risk in humans (Vainio et al., 1992). Prior to this, mechanistic and other relevant data had been used by IARC working groups on an *ad hoc* basis to inform overall evaluations of carcinogenicity, mainly to upgrade overall evaluations. Additional efforts to formalize the consideration of mechanistic evidence within the IARC classification system also occurred in the 2005-2006 timeframe during the most recent updating of the IARC preamble (Cogliano et al., 2008). As described in Cogliano et al. (2008), it is the IARC viewpoint that “Mechanistic data can be pivotal in IARC evaluations when the evidence in humans is not conclusive (that is, there is neither sufficient evidence nor evidence suggesting lack of carcinogenicity in humans).” Cogliano et al. (2008) highlighted the probative roles that mechanistic data have played in the raising and lowering of classifications for a variety of agents, often providing critical insights on the relevance of positive animal bioassays to humans.

Given this background on the IARC classification process, it can now be asked how the more recent health effects findings affect the weight of the evidence for DE carcinogenic potential, focusing first on implications for TDE carcinogenic potential. As discussed earlier, approximately 19 epidemiologic analyses of historical DE exposures and lung cancer risk have been published in the last 10 years, and a greater number since 1988. However, even the most recent studies have many of the same limitations and weaknesses as older studies. In particular, despite some improvements in study design, the majority of recent studies continue to show only small increased lung cancer risks among DE exposed populations, along with inconsistent evidence of an exposure-response relationship. That is, we may not have advanced much beyond the “large but equivocal body of epidemiologic evidence” described by Dr. Debra Silverman of the US National Cancer Institute in 1998 (Silverman, 1998).

Ward et al. (2010) proposed that the epidemiologic evidence on TDE would be significantly strengthened when the analyses for the NIOSH-NCI study of US underground miners were published. Although the Attfield et al. (2012) and Silverman et al. (2012) findings represent important contributions to the DE health effects literature, it is important to emphasize that they remain limited by an uncertain retrospective exposure assessment that relies on assumptions and predictions rather than actual DEP exposure measurements. As discussed previously, the causal implications of the DEMS findings are tempered by a number of inconsistent and unexplained findings; greater scrutiny of the voluminous body of statistical findings is needed to ensure their correct interpretation. Furthermore, the DEMS findings for mining populations are of uncertain relevance to other DE-exposed populations, given that historical DE exposures of miners were dominated by emissions from older diesel engines that were never fully characterized. As noted by HEI (1995), "large uncertainties are associated with applying emissions or exposure data from one type of engine during a specific time period to risk assessments for other populations and time periods.” In addition, the DEMS findings are of limited, if any, relevance to DE exposures in more contemporaneous mining environments where improvements in diesel engine technologies and mine ventilation, together with the implementation of more stringent emissions standards and fuel requirements, have contributed to reduced DE emissions (Mischler and Colinet, 2009).

With respect to the experimental, laboratory evidence, it has been previously mentioned that the most significant development since 1988 involves our improved understanding of the crucial role of the lung-clearance-overload mechanism in leading to the positive rat bioassay results. In particular, there is now a better understanding of the species-specific nature of this mechanism, its threshold dependence, and the fact that it is not DEP-specific and can result from prolonged and elevated exposures of rats to a variety of different inhaled-particle types. As discussed earlier, it is now widely accepted that the positive rat bioassay results were obtained under lung overload conditions and thus are not relevant to humans. Paired with IARC's greater emphasis on mechanistic data for informing car-cinogenicity classifications, it is expected that the progress related to the “rat-lung-overload” phenomenon will have implications on IARC's updated interpretation of the rat bioassay data. It is important to observe that IARC recently addressed the relevance of the rat-lung-overload phenomenon to humans during the 2006 reevaluation of carbon black (IARC, 2010). Despite the lack of evidence of a consistent excess of lung cancer among coal miners, IARC cited findings of high retained mass lung burdens and decreased lung clearance among coal miners as evidence of steps related to the lung-clearance-overload mechanism, concluding that “animal cancer data obtained under conditions of impaired lung clearance are relevant to humans” (IARC, 2010). During the June 2012 IARC meeting, another panel of experts will revisit this issue.

In contrast to TDE, there are currently few health effects data of relevance to the chronic exposure, carcinogenic potential of NTDE, although a chronic inhalation rat bioassay for NTDE is ongoing as part of the collaborative ACES efforts. There are no epidemiologic studies of direct relevance to NTDE and there may not be any for many years, not because populations have not been exposed to NTDE, but because historical exposures are entirely for TDE and current exposures continue to be a mixture of TDE and NTDE. There are currently available an abundance of emissions characterization data, as well as preliminary toxicological data, that distinguish NTDE from TDE. They demonstrate major reductions in numerous regulated and unregulated DE constituents in NTDE, chemical and physical changes to the DEP particle, and the elimination of some biological responses previously observed for TDE. These data are clearly not sufficient to support a hazard or cancer risk assessment for NTDE, but they provide scientific justification for the independent evaluation of TDE and NTDE hazards.

Clearly there is a need to better understand the carcinogenic potential of NTDE, with the ACES chronic bioassay expected to provide a number of key pieces of evidence. While there may remain uncertainties regarding the hazard and risk potential of NTDE, a sizable body of data demonstrates that today's NTDE should be viewed as a substance different from yesterday's TDE, just as TDE and GEE have always been considered to be different substances.
